# Chemo-Diversity and Secondary Metabolite Content in Corticolous Crustose Lichens (Fam. Arthoniaceae, Graphidaceae, Pyrenulaceae) from Remnants of Colombian Tropical Dry Forests

**DOI:** 10.3390/jof12070526

**Published:** 2026-07-17

**Authors:** Pierine España-Puccini, Amner Muñoz-Acevedo, Natalia A. Llanos-López, Marc Stadler, Mayar L. Ganoza-Yupanqui, Paula S. Burgos-Zelada, María C. Martínez-Habibe

**Affiliations:** 1 Department of Natural Sciences, Universidad del Norte, Puerto Colombia 080007, Colombia; pierine.espanap@unilibre.edu.co (P.E.-P.); mhabibe@uninorte.edu.co (M.C.M.-H.); 2 IMB—Research Group in Medicine and Biotechnology, Universidad Libre, Puerto Colombia 080007, Colombia; 3 Department of Microbial Drugs, Helmholtz Centre for Infection Research (HZI), 38124 Braunschweig, Germany; natalia.llanos-lopez@helmholtz-hzi.de (N.A.L.-L.); marc.stadler@helmholtz-hzi.de (M.S.); 4 Institute of Microbiology, Technische Universität Braunschweig, 38106 Braunschweig, Germany; 5 Medicinal Plant Quality Control Research Group, Faculty of Pharmacy and Biochemistry, Universidad Nacional de Trujillo, Trujillo 13011, Peru; mganoza@unitru.edu.pe (M.L.G.-Y.); pburgosz@unitru.edu.pe (P.S.B.-Z.)

**Keywords:** anthraquinones/xanthones, Caribbean region, chemotaxonomy, depsides, depsidones, dibenzofurans, lichen substances

## Abstract

Colombia ranks third among South American countries in terms of fungal diversity (including lichens); however, the lichen diversity of tropical dry forest ecosystems, particularly those in the Caribbean region, remains relatively understudied. The typical lichens found in tropical dry forest are crustose/corticolous microlichens (adapted to the xerophytic environments), which produce certain secondary metabolites that act as protective agents against ultraviolet radiation/decomposition/depredators; nonetheless, secondary metabolites of most of these lichens have not yet been described. This study focused on the compositional analysis and the extrolite content of seven lichens: *Cryptothecia* sp., *Cryptothecia scripta*, *Graphis dendrogramma*, *Leucodecton occultum*, *Helminthocarpon leprevostii*, *Pyrenula ochraceoflava*, and *Allographa seminuda*, using thin-layer chromatography, high-performance liquid chromatography–diode array detector, liquid chromatography–electrospray–mass spectrometry and/or liquid chromatography–tandem mass spectrometry. The main findings were: (i) two *Cryptothecia* spp. contained 2′-O-methylperlatolic acid (97%) or ovoic/gyrophoric acids (67%/28%); (ii) two specimens of *G. dendrogramma* had stictic/norstictic acids (33–60%/40%); (iii) three samples of *L. occultum* were characterized by norstictic acid (42–82%); (iv) schizopeltic/3-O-methylschizopeltic acids (58%/32%) were found in one specimen of *H. leprevostii*; (v) one sample of *P. ochraceoflava* was represented by unidentified xanthone-type metabolite/7-chloroemodin/ (26%/24%); and, (vi) the extrolite content in one specimen of *A. seminuda* was negligible. Finally, 10 of the 11 lichen specimens studied from the Department of Atlántico within Colombia contained certain secondary metabolites that likely reflect the common types of extrolites, namely, di-/tri-depsides, depsidones, dibenzofurans, and chlorinated anthraquinones/xanthones, which could be hypothetically related to the evolutionary patterns of this type of organism.

## 1. Introduction

Colombia, recognized for hosting almost 10% of the world’s biodiversity, is considered the third country with the highest wealth index and the second with the greatest diversity of vascular plants (24,025 species) [1]. The country’s biological richness is also reflected in its high diversity of fungi, including lichens, placing it third (5%—ca. 7250 spp.) among South American countries [2,3]. According to Moncada et al. [4], there are ca. 2700 species of lichenized fungi in Colombia, with the Andean region (including paramo ecosystems) harboring the greatest known richness of these organisms to date; in contrast, the lichen richness of Colombian tropical dry forest (TDF) ecosystems, particularly those in the Caribbean region, remains relatively understudied [5]. Nonetheless, it is also well-known that TDFs host a considerable diversity of microlichens (crustose lichens), particularly within families, such as Graphidaceae (order Graphidales), Arthoniaceae (order Arthoniales) and Pyrenulaceae (order Pyrenulales), which developed adaptations to xerophytic environments [6,7]. Correspondingly, the Graphidaceae family comprises crustose/corticolous lichens widely distributed in tropical ecosystems, recognized for producing some secondary metabolites (polycyclic acids) [6]. Meanwhile, lichens of the Pyrenulaceae family have perithecial ascomata (typically associated with smooth tree barks in humid environments), which contain pigments (from yellow to red) [8,9]. Finally, the Arthoniaceae family includes taxa with frequently cryptic thalli, producers of lichen substances derived mainly from orsellinic acid and orcinol [10].

Approximately 1610 secondary metabolites (characteristics, with molecular weights < 500 u) have been reported in lichens, some of which are recognized as pigments (e.g., yellow, orange, red and brown) that provide them with competitive advantages such as protection against ultraviolet radiation and decomposition [11,12,13]. Furthermore, these metabolites, due to their biological properties, could act as antibiotic/toxic, antifeedant, antiviral, antioxidant and anticancer agents [14,15,16]; depending on the main biogenic pathway (acetate–polymalonate), these can be anthraquinones, depsides, depsidones, dibenzofurans, xanthones, chromones, etc. [17,18,19]. Consequently, anthraquinones are characteristic compounds of lichen species, primarily from the family Teloschistaceae, while depsides are typical of lichens from the family Roccellaceae. Dibenzofurans, in turn, have been reported in the Cladoniaceae and Lecideaceae/Lecanoraceae/Roccellaceae families [9,20]. As reported by Stojanovic et al. [21], depsidones are widely distributed in several lichen families.

Regarding the three lichen families of the present study, the main components that have been reported in the scientific literature are: (i) depsidones (e.g., stictic, norstictic and protocetraric acids) for the Graphidaceae [22]; (ii) anthraquinones (e.g., parietin and emodin) for the Pyrenulaceae [23]; and (iii) depsides (e.g., confluentic and psoromic acids) for the Arthoniaceae [24]. Among these families, seven lichen species were selected for this study, namely *Allographa seminuda* Lücking & Kalb (Graphidaceae), *Graphis dendrogramma* Nyl. (Graphidaceae), *Leucodecton occultum* (Eschw.) Frisch (Graphidaceae), *Pyrenula ochraceoflava* (Nyl.) R.C. Harris (Pyrenulaceae), *Helminthocarpon leprevostii* Fée (Arthoniaceae), *Cryptothecia scripta* (Arthoniaceae), and *Cryptothecia* sp. (Arthoniaceae), which are abundant and frequent, but with limited biomass (inherent to the growth biotype), in the TDFs of the Department of Atlántico.

Nonetheless, none of these have been chemically characterized by thin-layer chromatography/liquid chromatography–mass spectrometry (TLC/LC–MS), nor has their chemical composition been reported. This knowledge gap is particularly significant considering that the environmental conditions of the TDF—high solar irradiation and temperature, seasonal water deficit, and diversity of phorophyte substrates—can act as determining factors in the biosynthetic regulation of secondary metabolites, potentially favoring the production of unique compounds or the configuration of eco-geographically differentiated chemotypes, a phenomenon evidenced in lichens from other regions of the world [25,26]. Therefore, this study focused on determining the lichen substance content and chemical composition of eleven extracts from seven species of crustose lichens belonging to the families Arthoniaceae, Pyrenulaceae and Graphidaceae by thin-layer chromatography (TLC), high-performance liquid chromatography–diode array detector (HPLC–DAD), liquid chromatography–electrospray ionization–mass spectrometry (LC–ESI–MS) and liquid chromatography–tandem mass spectrometry (LC–MS/MS) (or direct infusion–mass spectrometry—DI–MS/MS) analysis.

## 2. Materials and Methods

### 2.1. Lichen Samples

**Figure 1:**
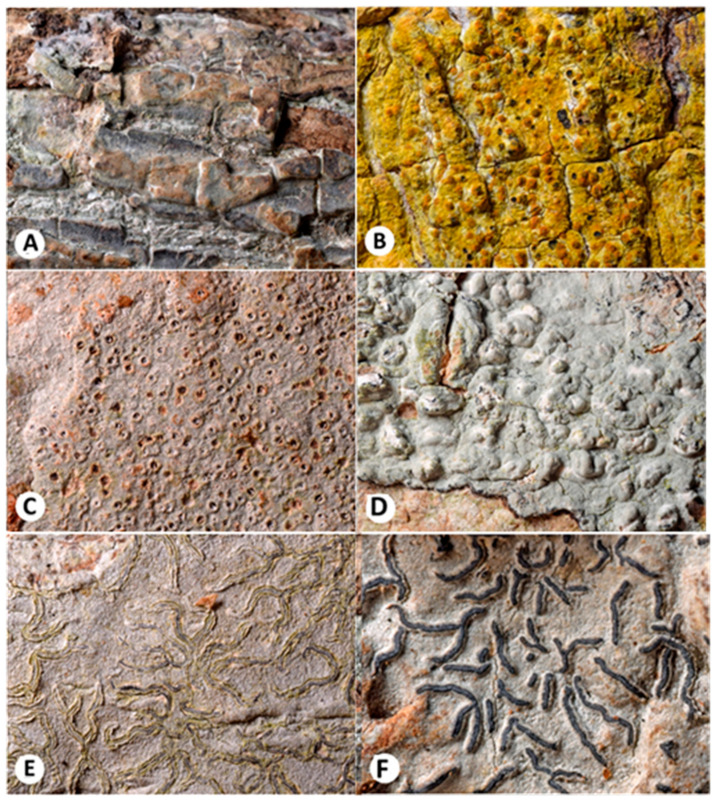
Some specimens selected for this study: (**A**) *Cryptothecia* sp. (CM3094b; C-SP1); (**B**) *Pyrenula ochraceoflava* (CM3136; P-OCH); (**C**) *Leucodecton occultum* (CM3545; L-OCU1); (**D**) *Helminthocarpon leprevostii* (CM3465; H-LEP); (**E**) *Graphis dendrogramma* (CM3453; G-DEN1); and (**F**) *Allographa seminuda* (CM3010; A-SEM).

Eleven well-developed lichen specimens were selected from five remnants of tropical dry forests in northern Colombia (department of Atlántico), representing three classes, three families, six genera, and seven species (Table 1 and Figure 1), from the previously collected specimens inventoried by España-Puccini et al. [7], where the sampling strategy was also described in detail. The seven taxa corresponded to common species (abundant and frequents) in this ecosystem. The biological samples were supplied by Dr. Robert Lücking (Botanischer Garden and Botanisches Museum BGBM, Germany) and corresponded to Colombian specimens catalogued in the BGBM collection.

For clarity, the specimens studied were not randomly chosen as single representatives of each species; instead, they were selected after chemical screening by TLC [7]. This analysis allowed for comparison of the chemical profiles within each specimen of a species to identify whether they exhibited the same TLC patterns; when specimens showed congruent TLC patterns (same spots), they were analyzed by HPLC–DAD to confirm the similarity of their constituents, based on retention times (R_t_) and UV–Vis spectra, and then, one specimen with sufficient biomass was selected for detailed/robust chemical analysis. In contrast, when TLC/HPLC–DAD revealed different chemical profiles among specimens assigned to the same species, these specimens were treated separately and selected for subsequent robust analysis.

### 2.2. Extraction Procedure

Once the lichen samples were taken to the processing laboratory, each sample was prepared by carefully removing the thallus from the remaining tree bark using classic mini steel-wire brushes (mechanical removal technique) (Rolson, Quality Tools 7, Twyford, Berkshire, UK; Figure S1). Then, 1–2 g of each material (dehydrated) and 3–6 mL of the extraction mixture (1:1 ratio, methanol:ethyl acetate–ACS grade solvents, Merck, Darmstadt, Germany) were added into a test tube and subjected to sonication for 2 h at 40 °C (this procedure was performed three times per lichen); afterward, each combined extract was filtered, concentrated, and analyzed by TLC, HPLC–DAD, LC–HRMS (ESI) and/or LC–MS/MS. Finally, two depsidones (stictic and constictic acids) previously isolated and chemically characterized by España et al. [27], from *G. dendrogramma* (CM3453) extract, were used as standards to compare retention times (R_t_—HPLC), mass spectra (fragmentation patterns) and UV–Vis spectra for the depsidones.

### 2.3. Chemical Analysis

#### 2.3.1. Thin-Layer Chromatography

Each extract (30 µg/mL) was subjected to TLC analysis, as reported by España et al. [27], based on procedures described by Culberson and Kristinsson [28] and Schumm and Elix [29], using Merck aluminum silica gel 60 F_254_ TLC plates (20 × 20 cm; Darmstadt, Germany) and solvent mixtures A (toluene:dioxane:glacial acetic acid–ACS grade solvents, Merck, Darmstadt, Germany) and C (toluene:glacial acetic acid). In addition, spots on the TLC plates were developed and visualized using UV–Vis lights and solutions of H_2_O, H_2_SO_4_ or KOH as chemical developers. The color, intensity, and relative retention factor (R_f_) values of the spots were qualitative criteria for assigning the presumptive identity of the lichen substances [29,30]. Likewise, *Parmotrema perforatum* (Jacq.) A. Massal. (USA, Florida, Streimann 40118, B), which contains atranorin and norstictic acid, was used as a TLC control for lichens. Finally, the TLC images were obtained by digital photographic recording of the TLC plates after their development/reveal.

#### 2.3.2. High-Performance Liquid Chromatography–Diode Array Detector

Each extract (30 µg/mL) was analyzed using an Ultimate 3000 UHPLC system (Dionex—Thermo Fisher Scientific; Waltman, MA, USA) equipped with a diode-array detector (DAD, UV–Vis—190–600 nm) and a Capcell-Pak^®^ C_18_ UG120 column (120 Å, 250 mm × 4.6 mm (i.d.) × 5 μm (p.s.); Shiseido Co, Ltd., Tokio, Japan). The analytical parameters were those as reported by Feige et al. [31] and Yoshimura et al. [32]. Chromatographic data were processed with Chromeleon^®^7 Chromatography Data System software (version 7.2.1.5833, Thermo Fisher Scientific; Waltman, MA, USA). Blanks of extraction, solvents, and column were performed, as well as the analysis of some tree bark extracts (from where the lichens were collected) to rule out cross-contamination or interference.

#### 2.3.3. Liquid Chromatography–Electrospray Ionization–Mass Spectrometry

Electrospray ionization mass spectrometry data were recorded using an amaZon^®^ speed ESI–Iontrap–MS (Bruker; Billerica, MA, USA) coupled to an UltiMate^®^ 3000 Series UHPLC (Thermo Fisher Scientific; Waltman, MA, USA) equipped with a C_18_ Acquity^®^ UPLC BEH column (2.1 mm (i.d.) × 50 mm, 1.7 μm (p.s.); Waters Corporation, Milford, MA, USA) and a DAD (190–600 nm). The analysis was carried out under the conditions reported by Llanos-López et al. [33] and included blanks of extraction, solvents and column. The mass spectra of lichen metabolites were compared with those from published data, available databases such as The Natural Products Atlas (https://www.npatlas.org/) and Lichen MS/MS Database (https://gnps.ucsd.edu/ProteoSAFe/gnpslibrary.jsp?library=LDB_POSITIVE (accesed on 10 november 2024); https://gnps.ucsd.edu/ProteoSAFe/gnpslibrary.jsp?library=LDB_NEGATIVE (accesed on 20 may 2025))], along with the specialized literature [34,35,36]. The relative percentages of each component detected by HPLC–DAD/MS (as a semiquantitative measure) for each lichen extract were calculated using the area normalization method $\left[\left(\frac{Metabolite\;peak\;area\;}{Sum\;of\;all\;metabolite\;peak\;areas}\right)\times 100\right]$, under the assumption that the response factors were similar for compounds containing the same type of structure.

#### 2.3.4. Liquid Chromatography–Tandem Mass Spectrometry

MS/MS spectra for some extracts (30 µg/mL for extract) were obtained by a Xevo TQ-XS triple quadrupole mass spectrometer (Waters Corporation, Milford, MA, USA) coupled to an Acquity^®^ Arc System UHPLC (Waters Corporation, Milford, MA, USA) with an Acquity^®^ UPLC BEH C_18_ column (2.1 mm (i.d.) × 100 mm × 1.7 μm (p.s.); Waters Corporation, Milford, MA, USA) and a precolumn filter (40 °C), and an ESI source and a photodiode-array detector (PDA, 200–600 nm). In both positive and negative ESI modes, the survey scan function was applied using Scan Wave MS and daughter scan (mass range: *m*/*z* 50–1500; collision energy: 15/30 eV). Spectral acquisition and processing were carried out with MassLynx software (version 4.2, Waters Corporation, Milford, MA, USA). The other analytical conditions were applied as reported by Torres-Guevara et al. [37] with modified conditions of elution (Phase A: H_2_O:0.1% HCOOH; Phase B: MeCN: 0.1% HCOOH) (LiChrosolv solvents, Merck, Darmstadt, Germany): 0–0.21 min (70% A), 3.13 min (30% A), 6.25 min (0% A), 8.34 min (0% A), 9.80 min (70% A), and 12.00 min (70% A). A flow rate of 220 μL/min and an injection volume of 1 μL. Additionally, some extracts (30 µg/mL) were characterized by direct infusion mass spectrometry (UHPLC–DI–ESI–MS/MS) under the following setting: daughter scan mode, continuum data format, 1 min (duration), 1 s (scan time) and *m*/*z* 100–450 (mass range).

### 2.4. Cluster Analysis

The raw data of the percentage chemical composition (input matrix) of all analyzed lichen extracts were subjected to cluster analysis (CA) (by OriginPro 2025b SR1, v. 10.2.5.234) as an exploratory visualization tool relating chemical composition to lichen species. For this purpose, the “group average” was used as the linkage method, along with the Euclidean distance as the distance metric (using normalized min-max variables between 0 and 1) and grouping based on “similarity”. Although the dendrogram axis was displayed as similarity values in OriginPro, the clustering itself was based on Euclidean distances. Nevertheless, the similarity values were generated by OriginPro from the Euclidean distance values using the transformation S = 100 × (1 − d/d_max_), where d is the distance at a given clustering stage and d_max_ is the maximum distance observed at the clustering stages. Therefore, the dendrogram represented a hierarchical distance-based clustering, visualized on a transformed similarity scale.

## 3. Results

**Figure 2:**
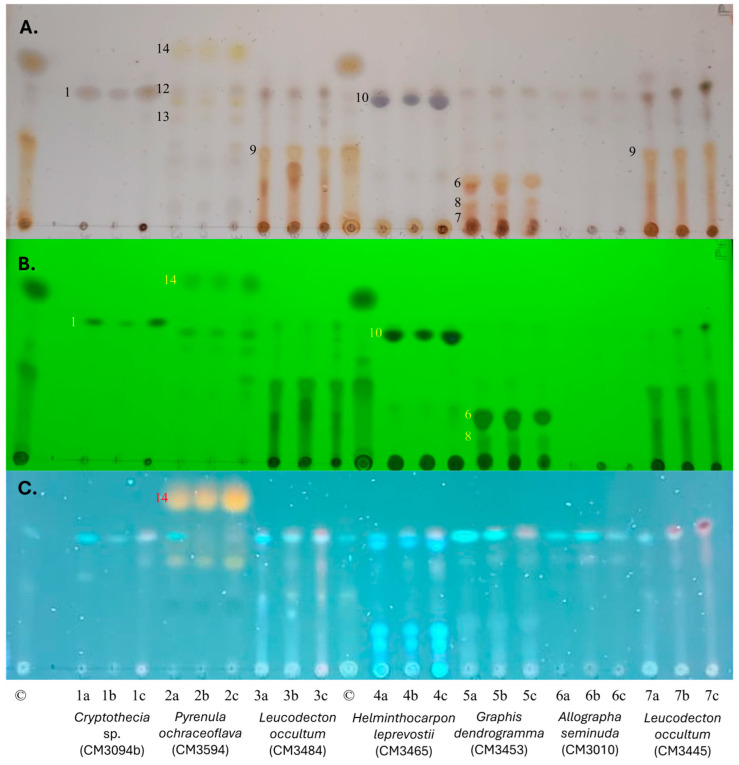
TLC analysis of seven lichen extracts. Each sample was prepared in triplicate, using three different techniques to remove the lichen thallus: (a) coarse mechanical removal including bark fragments; (b) standard technique of mechanical removal by scraping the surface of the lichen thallus with a razor blade; and (c) refined technique of careful mechanical removal using mini wire brushes (Figure S1). (**A**) TLC plate after development with 10% sulfuric acid and heating, under visible light; (**B**) under short-wave UV light (254 nm); and (**C**) under long-wave UV light (366 nm). The numbers correspond to the following compounds: ©. control, 1. 2′-O-methylperlatolic acid, 6. stictic acid, 7. constictic acid, 8. cryptostictic acid, 9. norstictic acid, 10. schizopeltic acid, 12. 7-chloroemodin, 13. emodin, and 14. parietin (+unknown compound).

For the presentation of results/discussion, each lichen sample was labeled with a reference acronym, i.e., *Cryptothecia* sp. (C-SP1, Usiacurí), *Cryptothecia* sp. (C-SP2, Usiacurí), *C. scripta* (C-SCR), *Graphis dendrogramma* (G-DEN1, Tubará), *G. dendrogramma* (G-DEN2—Luruaco), *Helminthocarpon leprevostii* (H-LEP), *Leucodecton occultum* (L-OCU1, Tubará), *L. occultum* (L-OCU2, Piojó), *L. occultum* (L-OCU3, Luruaco), *Pyrenula ochraceoflava* (P-OCH), and *Allographa seminuda* (A-SEM). Then, the chemical screening of the extracts began using TLC analysis (considering the R_f_ values in solvent mixture C—Figure 2 and Figure S3), and, subsequently, was complemented by LC–DAD/MS (ESI) analysis. The chromatograms and presumptive chemical composition are shown in Figure S2 and Table S2, respectively; these results are presented lichen by lichen.

Thus, one spot was detected in the TLC analysis for the two *Cryptothecia* sp. extracts, which, based on its color (UV–Vis light) and R_f_ value (63), was tentatively identified as 2′-O-methylperlatolic acid (**1**-didepside) (Figure 2 and Table S2); nonetheless, three components [2′-O-methylperlatolic acid (**1**) and isohyperplanaic acid (**2**), along with an unidentified component (protonated/deprotonated molecular ions—[M + H]^+^/[M − H]^−^: 593.3254/591.3210)] were detected by LC–MS analysis. The two putatively identified depsides [**1** (~97%), and **2** (~2–3%),] almost certainly contributed ca. 99–100% to the total extract composition. In addition, the chromatographic (HPLC) and spectroscopic (UV–Vis) data obtained for the main constituent (**1**) were: R_t_ (min): 14.84; λ_UV_ (nm): 221 (max), 271, 308.


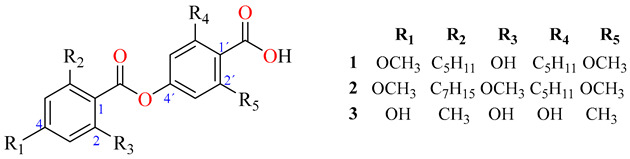


In contrast, in the TLC analysis of *C. scripta* extract, one stained/observed spot, was observed, which, according to the R_f_ value (33), could be gyrophoric acid (**4**-tridepside); conversely, LC–MS analysis showed that the lichen extract contained four constituents: ovoic acid (**5**-tridepside), gryrophoric acid, lecanoric acid (**3**) and one unidentified component ([M + H]^+^/[M − H]^−^: 333.0391/330.8974). The three presumptively identified metabolites [**4** (~28%), **5** (~67%), and **3** (~4%),] possibly contributed ca. 99% to the total extract composition. Moreover, the chromatographic and spectroscopic (UV–Vis) data for the main constituents were: (**4**)—R_t_: 10.42 min, λ_UV_: 226 nm, 269 nm (max), 304 nm; (**5**)—R_t_: 10.51 min, λ_UV_: 231 nm, 269 nm (max), 305 nm. The structural difference between (**4**) and (**5**) is based on the substitution at C-2′: (**4**) and (**5**) contain, respectively, the -OH and -OCH_3_ groups.


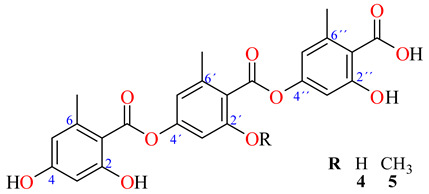


Considering two specimens of *Graphis dendrogramma*, depsidones, such as stictic acid (**6**-~60%), constictic acid (**7**-~27%), and cryptostictic acid (**8**), etc., were the main constituents tentatively identified in the *G. dendrogramma* (G-DEN1, from Tubará) extract by both TLC and LC–MS; then, depsidones (**6**) and (**7**) were isolated and structurally characterized to confirm their structures [27]. In the case of another *G. dendrogramma* specimen (from Luruaco, G. DEN2), TLC analysis of the extract revealed three intense spots (Figure 2), with R_f_ values (Table S2) matching for three depsidones: norstictic acid (**9**), stictic acid, and constictic acid.


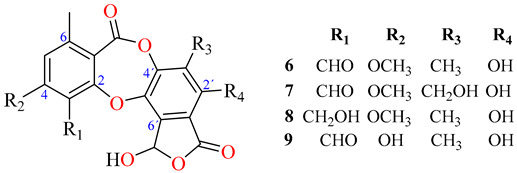


Nonetheless, LC–MS analysis indicated that 13 major substances constituted the extract, of which three [norstictic acid (~40%), stictic acid (~33%), and constictic acid (~5%)] probably contributed ca. 78% to the total extract composition; other depsidones (based on the MS fragmentation pattern and UV–Vis spectra) contributed ca. 8%. The remaining constituents were not identified (ca. 14%). In addition, the chromatographic and UV absorption data for norstictic acid were, respectively, 8.36 min and 221 nm, 241 nm (max), 311 nm.

Similar to *Graphis* sp., three samples of the species *Leucodecton occultum*, studied from different locations in the Department of Atlántico, showed a high content of depsidones based on TLC and LC–MS analysis. Thus, the TLC analysis of total extracts from *L. occultum* (L-OCU1, L-OCU2 and L-OCU3) from different locations (CM3545—Usiacurí, CM3484—Piojó, and CM3661—Luruaco) showed similarities in the spots observed (Figure 2), both in their colors (yellow to brown)/intensities and in R_f_ values, although a more intense spot (R_f_: 63) appeared in TLC of L-OCU1; these spots tentatively corresponded to depsidones such as norstictic acid (**9**) and stictic acid (**6**).

The LC–MS analysis of the three extracts (Figure S2—L-OCU1, L-OCU2 and L-OCU3) revealed that they contained, respectively, 11, 20 and 6 components, and showed some similarities and notable differences, viz., the main constituent for them was probably norstictic acid (**9**), which differed in relative amounts (~46%—L-OCU1, ~42% L-OCU2, ~82% L-OCU3); the second constituent was presumptively stictic acid (~35%/~9%) and an unknown compound (~10%) for L-OCU1/L-OCU3 and L-OCU2, correspondingly; stictic acid (~7%) was tentatively the third constituent for L-OCU2. Finally, the content of depsidones/unknown compounds for three specimens was potentially ~97%/~3% (L-OCU1), ~60%/~40% (L-OCU2), and ~99%/~1% (L-OCU3).

On the other hand, the *H. leprevostii* extract exhibited six spots (with different colors, from greyish to violet-blue) in its TLC chromatogram when a chemical developer (H_2_SO_4_ solution) or UV–Vis light was applied (Figure 2) and, based on the R_f_ value (59) of the main colored spot (blueish), a dibenzofuran was preliminarily identified as schizopeltic acid (**10**); a second major spot (yellow-brown), partially overlapping the blueish spot, had an R_f_ value of 62. LC–ESI–MS analysis of the extract showed that it consisted of seven lichen substances (Table S2 and Figure S2) possibly distributed as ca. 90% dibenzofurans and ca. 10% unidentified compounds; the main constituents could be schizopeltic acid (**10**, ~58%) and 3-O-demethylschizopeltic acid (**11**, ~32%), whose structures differ in the substitution at C-3; i.e., (**10**) has the -OCH_3_ group, while (**11**) has the -OH group. The R_t_ of 8.49 min and 9.75 min, and wavelengths of 219–214 nm, 241–242 nm (max), 272–273 nm, 312–313 nm, were, respectively, the chromatographic and UV absorption data for these putative dibenzofurans.


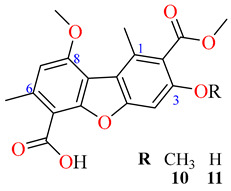


Regarding the chemical composition of the total extract of *P. ochraceoflava* analyzed by TLC, six to seven spots (of different colors, from yellow to brown) were exposed in its chromatogram when a chemical developer was applied (Figure 2); based on the R_f_ values (61, 48, and 74), three anthraquinones (main colored spots—yellowish to orange) were provisionally identified as 7-chloroemodin (**12**), emodin (**13**) and parietin (**14**). However, LC–ESI–MS analysis of the extract indicated that it was composed of at least 19 lichen substances (Table S2 and Figure S2), including some unidentified constituents (~61%); however, despite this high percentage of unknown metabolites, they could be categorized into a family of compounds according to the MS fragmentation pattern and UV–Vis spectra. Therefore, ca. 38% of the constituents were tentatively identified as anthraquinone derivatives, of which ca. 26% were chlorinated derivatives. In addition, ca. 26% were xanthone derivatives, while ca. 1% were depsidones and ca. 35% were non-categorized unknown compounds. Consequently, the presumptively identified components were 7-chloroemodin (**12**, ~24%), emodin (**13**), parietin (**14**), and norstictic acid (**9**). Additionally, the chromatographic and spectroscopic (UV–Vis) data for these constituents were: (**12**)—R_t_: 10.87 min, λ_UV_: 225 nm (max), 274 nm, 290 nm, 310 nm, 436 nm; (**13**)—R_t_: 10.09 min, λ_UV_: 225 nm (max), 266 nm, 291 nm, 437 nm; and (**14**)—R_t_: 12.22 min, λ_UV_: 225 nm (max), 268 nm, 290 nm, 437 nm.


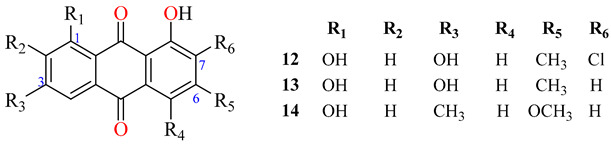


It is noteworthy that the most abundant lichen substance (~26%) of the extract could not be identified; however, its protonated/deprotonated molecular ions ([M + H]^+^/[M − H]^−^: 302.9630/300.9250) allow for a molecular formula of C_16_H_14_O_6_ (M_W_: 301.9440 u) to be suggested, and, based on its MS fragmentation pattern and UV–Vis spectrum, the component could be a xanthone-like metabolite (yellowish, λ: 254 nm, 303 nm and 366 nm). Likewise, three other unidentified lichen substances, present in high proportions (~12%, ~7% and ~10%), had molecular ions ([M + H]^+^/[M − H]^−^) at *m*/*z* 465.2958/463.2998 (probable molecular formula: C_27_H_28_O_7_, M_W_: 464.2978 u), *m*/*z* 449.3272/447.2938, and *m*/*z* 449.3127/447.2893, respectively. In addition to having similar molecular ions, the latter two substances showed the same UV–Vis spectra (λ_UV_: 226 nm (max), 319 nm), suggesting that they might be isomers.

Finally, the *A. seminuda* extract showed no spots on the TLC chromatogram; only one component was detected (negligible) by LC–MS, which could not be identified. Table 2 contains detailed information related to the LC–MS (ESI–Iontrap) analysis of the two constituents with the highest relative percentage amounts, tentatively identified in the extracts of each lichen studied. In addition, the Table includes the other methods used as analytical tools to support the putative identification.

In reference to the cluster analysis, a hierarchical cluster tree was plotted for the chemical composition data of ten specimens studied in this work (excluding *A. seminuda*, since no secondary metabolite was detected in this lichen with the analytical tools available/used) and including tentative compositional data from the three specimens reported by España et al. [27]. The chemical composition criteria were the probable content of di-/tridepsides, stictic acid-related depsidones, protocetraric acid-related depsidones, dibenzofurans and chlorinated–(or non)–antraquinones/xanthones.

**Figure 3:**
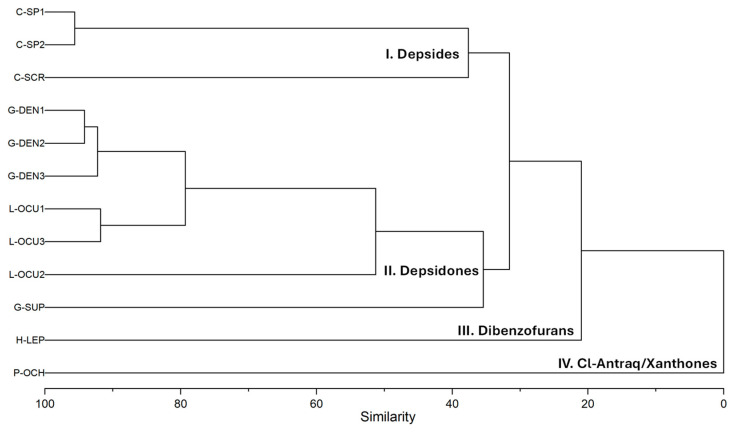
Hierarchical tree chart of all studied lichen specimens from the Department of Atlántico (Colombia), based on the determined chemical composition.

As a result, four main clusters were recognized in the hierarchical tree (Figure 3): cluster I—joined to two *Cryptothecia* spp., composed of depsides (di-/tri-; ca. 98–100%); cluster II—linked to *G. dendrogramma* (three specimens) (ca. 88–97%), *L. occultum* (three specimens) (ca. 64–99%) and one sample of *G. supracola* (ca. 90%), containing depsidones (stictic acid complex and protocetraric acid complex); cluster III—related to one specimen of *H. leprevostii*, probably rich in dibenzofurans (ca. 90%); and, cluster IV—associated to one sample of *P. ochraceoflava*, with its presumptive content of chlorinated anthraquinones/xanthones (ca. 76%). Then, the hypothetical distribution of secondary metabolites in the studied specimens (lichen population) was as follows: 54.5% for depsidones, 27.3% for depsides, 9.1% for dibenzofurans, and 9.1% for anthraquinones/xanthones.

## 4. Discussion

This study focused on the presumptive chemical composition of eleven crustose lichen specimens, revealing a plausible structural diversity of lichen substances in their extracts. Again, the application of TLC and LC–MS, as well as UV–Vis spectra (as a complementary analytical tool), was useful in supporting the structural elucidation of most of the putatively identified secondary metabolites. For those that were not fully identified, it allowed for the suggestion of possible chemical scaffolds (type of compounds).

Considering *Cryptothecia* spp., it should be noted that the lichen C-SP1 (and C-SP2) is possibly an unidentified/undescribed species to science because it differs morphologically (e.g., ascospore size) from the other species previously described for the genus; therefore, its presumed chemical composition is reported for the first time. Likewise, the lichen substance content (chemical profile: two didepsides; 2′-O-methylperlatolic acid as the main component) for this species (potentially undescribed *Cryptothecia* sp.) was the same for the two specimens collected from different locations, possibly suggesting that both lichen samples are found in similar ecological environments; however, this speculative interpretation of an ecological nature would require further studies for its verification. Then, upon reviewing the scientific literature, it was found that 2′-O-methylperlatolic acid (the probable constituent of the C-SP extracts) has been present in other *Cryptothecia* spp., e.g., *C. candida* s. str., *C. albomaculans*, *C. culbersoniae*, *C. galapagoana* and *C. evergladensis*, which could indicate that (**1**) is one of the biomarkers for species of this genus [38,39,40,41].

**Scheme 1:**
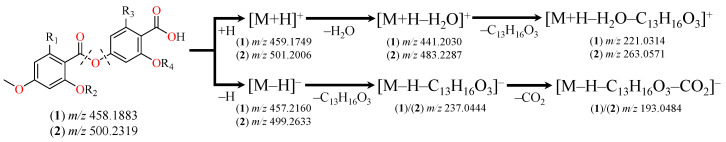
Suggested fragmentation route for depsides analyzed by MS/MS.

The structural analysis of (**1**) was based on UV–Vis and mass spectra; in consequence, the UV–Vis spectrum showed characteristic absorption bands at 221 nm (λ_max_), 271 nm, and 306 nm (Figure S4A), as mentioned above, coinciding with those reported by Huneck and Schreider [42], and Halıcı et al. [43]. Furthermore, the mass spectrum (Figure S5A), showed that the main fragments for (**1**) in negative mode were the ions at *m*/*z* 237.0444 and *m*/*z* 193.0484, speculatively corresponding to structures related to O-methylolivetol-carboxylic acid [C_13_H_17_O_4_]^−^ and olivetol monomethyl ether [C_12_H_17_O_2_]^−^, respectively, governed by alpha cleavage at the C=O (or -O-) function of the ester group [44,45,46], and complemented by decarboxylation. Meanwhile, the fragment at *m*/*z* 441.1889 corresponded to the loss of one H_2_O molecule (*m*/*z* 17.9793) from [M + H]^+^ (*m*/*z* 459.1682) (Scheme 1), and the elimination of the C_13_H_16_O_3_ fragment produced the ion at *m*/*z* 221.0572.

Likewise, the mass spectrum of (**2**) showed a similar fragmentation pattern in both modes; i.e., alpha cleavage at the carbonyl function of the deprotonated MI produced the ions at *m*/*z* 237.0287 and *m*/*z* 193.0421, while the protonated MI (*m*/*z* 501.1897), by loss of one H_2_O molecule and elimination of C_13_H_16_O_3_, generated the ions at *m*/*z* 483.1250 and *m*/*z* 263.0824, respectively.

MS/MS analysis (Figure S6) of the deprotonated molecular ion (*m*/*z* 457.1834) from the depside (**1**) showed five product ions at *m*/*z* 193.0484 (100%), *m*/*z* 236.9945, *m*/*z* 178.0688, *m*/*z* 297.9827, and *m*/*z* 121.9543; nevertheless, the first two fragments matched those described above but differed in their intensities. In contrast, the ions at *m*/*z* 178.06888 ([C_11_H_14_O_2_]^−^) and *m*/*z* 121.9543 ([C_7_H_6_O_2_]^−^) were hypothetically generated from the base peak ion [C_12_H_17_O_2_]^–^ (olivetol monomethyl ether) due to the cleavage of the -CH_3_ (*m*/*z* 14.9796) and -C_5_H_11_ (*m*/*z* 71.0941) groups, correspondingly.

It is worth highlighting that when LC-MS analysis was performed on the C-SP extracts, and the molecular weight (458.1637) and chemical formula (C_26_H_34_O_7_) of the main metabolite (tentatively 2′-O-methylperlatolic acid) were established, three other didepsides (e.g., 2-O-methylperlatolic acid [positional isomer—substitutions at C-2 (-OCH_3_) and C-2′ (-OH)], 4-O-demethylplanaic acid and 2,2′-di-O-methylanziaic acid) had the same molecular mass/chemical formula, according to the consulted literature [34,35,36]; however, their R_f_ values (particularly in solvent mixture C) differed from the values for (**1**) (both from the literature and experimental results). Therefore, conventional TLC analysis, although considered a screening technique, proved to be a discriminatory/complementary tool (to MS) for assigning the possible identity of this didepside.

Lastly, the depside 2′-O-methylperlatolic acid (as a major or secondary component) is a secondary metabolite found/identified in several lichen species, e.g., in *Buellia densipruinosa*, *B. stellulata*, *Chicitaea yueliangshanensis*, *C. assateaguensis*, *C. confuse* (basionym *Loxospora confuse*), *C. lecanoriformis* (basionym *Loxospora lecanoriformis*), *C. cristinae* (basionym *Loxospora cristinae*), *Enterographa zonata*, *Flavonora stramineoalbida* (basionym *Lecanora stramineoalbida*), *Inoderma applanatum*, *I. afromontanum*, *I. byssaceum*, *I. platygraphellum*, *I. sorediatum*, *Immersaria aegaea*, *Lecanora paramerae*, *L. achroa*, *L. helva*, *Lecidea tibetica*, *L. plana*, *Lecidea* sp., *Myriostigma candidum*, *Opegrapha* cf. *arengae*, *Pertusaria atroguttata, P. complanata*, *P. mesotropa*, *P. pertractata*, *P. submaritima*, *P. werneriana,* and *Stirtonia macrocarpa*, from different places in the world (e.g., Bolivia, Brazil, China, Pakistan, South Korea, Europe, Australasian Region, etc.) [47,48,49,50,51,52,53,54,55,56,57,58,59,60,61,62,63,64,65].

On the other hand, during the taxonomic identification of a Colombian *C. scripta* sample, an interesting exchange of views arose related to its identity due to the similarity with *C. striata*, based on the morphological and microscopic characteristics, as well as the TLC analysis of its lichen substances; both lichens (their extracts) showed one strong spot, with R_f_: 33 corresponding to gyrophoric acid (**4**), which is a chemo-marker for *C. striata*, according to Thor [66] and Bungartz et al. [40]. However, when LC–MS analysis was performed on this lichen extract, two chromatographic peaks (almost overlapping) appeared (R_t_: 10.42 min, 10.51 min—Figure S2) along with two different mass spectra/molecular weights (468.0335 u and 481.9944 u), corresponding probably to gyrophoric acid (**4**) and ovoic acid (**5**), respectively. Subsequently, upon reviewing the taxonomic key in the scientific literature based on this finding, the species containing the two tridepsides is *C. scripta*, as indicated by Elix [67]. The aforementioned misunderstanding arose because gyrophoric and ovoic acids have the same R_f_ value by TLC when solvents A and C are used [30,68,69], and, therefore, only a single spot would be visible on the TLC. This observation is pertinent because, in most cases, classical TLC analysis is the most widely used tool for lichen taxonomy (due to its practicality and cost), and, in some cases, it could lead to taxonomic inaccuracies (as mentioned above); nevertheless, due to the limitations that could appear in TLC, it is always advisable to additionally use robust separation techniques (e.g., gas and/or liquid chromatography) accompanied by spectroscopic methods (MS, UV–Vis, etc.).

**Scheme 2:**
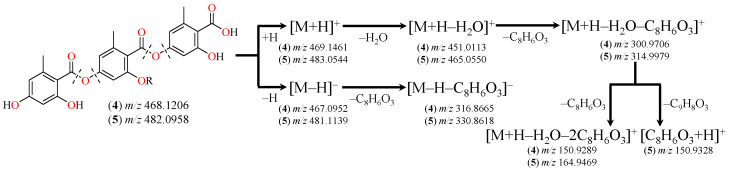
Proposed fragmentation pathway for the tridepsides analyzed by ESI–MS.

The structural analysis based on UV–Vis and mass spectra for gyrophoric (**4**) and ovoic (**5**) acids was as follows: the characteristic UV–Vis absorption bands [226–231 nm, 269 nm, and 304–308 nm (Figure S4B,C)] for both tridepsides were consistent with those reported by Zlatanović et al. [70], Meeßen et al. [71], Goga et al. [72], Kosanić et al. [73], Rao et al. [74], and Huneck et al. [75], although with slight differences (a few units) in the wavelength values. Likewise, the possible fragmentation pattern of these molecules was governed by the breaking of the depside bonds (Scheme 2) described by Huneck et al. [44]. Thus, after the molecular protonation process of (**4**)/(**5**), both molecular ions ([M + H]^+^) produced plausible fragments by the elimination of one H_2_O molecule and two successive losses of orsellinaldehyde units (*m*/*z* 150.0407—derived from orsellinic acid) at *m*/*z* 451.0113/*m*/*z* 465.0550, *m*/*z* 309.9706/*m*/*z* 314.9979, and *m*/*z* 150.9289/*m*/*z* 164.9469, respectively.

Nonetheless, an additional ion ([C_8_H_6_O_3_ + H]^+^) was observed at *m*/*z* 150.9328 in the mass spectrum of (**5**), which would result from the loss of the 2-O-methylorsellinaldehyde unit from the depside-derived fragment at *m*/*z* 314.9979. On the other hand, after the molecular deprotonation process of (**4**)/(**5**), both molecular ions ([M − H]^−^) generated fragments at *m*/*z* 316.8665/*m*/*z* 330.8618, speculatively by the elimination of an orsellinaldehyde unit (*m*/*z* 150.0407), promoted by an alpha cleavage at the ester group. The negative-mode mass spectrum of gyrophoric acid was like that reported by Mohammadi [76].

Comparison of these probable tridepsides with the available scientific literature indicated that: (i) both tridepsides are typical of the family Umbilicariaceae and the genus *Umbilicaria* (mainly gyrophoric acid), and (ii) ovoic acid is part of the gyrophoric acid chemosyndrome. Accordingly, Feige et al. [77] studied 11 species of *Umbilicaria* (93 specimens), which contained primarily gyrophoric acid, some of which also contained ovoic acid (later identified in these samples). Similarly, Seriña et al. [69] reported that Spanish species of *Umbilicaria* (e.g., *U. torrefacta*, *U. decussata*, *U. proboscidea*, *U. havaasii—*32–182 specimens) contained gyrophoric and/or ovoic acids in varying percentage values (61–90%**—4**)/(3–28%**—5**). Culberson et al. [68] identified gyrophoric (major) and ovoic (moderate) acids in *Dimelaena oreina* (Caliciaceae). Finally, from *Melanelia substygia* (basionym *Parmelia substygia*, fam. Parmeliaceae), Huneck et al. [75] isolated ovoic acid for the first time.

With reference to *Graphis dendrogramma* (G-DEN1 and G-DEN2) from different locations (Tubará and Luruaco) in the Department of Atlántico, both lichen samples consisted of depsidones belonging to the stictic acid complex, although they presented differences, i.e., G-DEN1 contained mainly stictic acid (**6**)/constictic acid (**7**), while G-DEN2 contained norstictic acid (**9**). However, when comparing the chemical composition of G-DEN2 (Luruaco) with that of the G-DEN (Piojó) previously described by España et al. [27], although both lichens presumptively contained norstictic acid, notable differences were observed in the relative amounts and in the other tentatively identified components. Specifically, the presumed content of norstictic/stictic acids was 40%/33% and 72%/10%, respectively, for G-DEN2 and G-DEN (Piojó). Surprisingly, both specimen sampling sites are considered areas with low human disturbance (private protected zones). Nonetheless, the lichens were collected at different times and years (G-DEN2: October 2023, rainy season; G-DEN (Piojó): December 2021, dry season), which could affect (increase or slow down) the production of secondary metabolites, possibly due to their response to season-dependent changes such as hydration period, irradiance, and temperature [78,79]. This interpretation remains hypothetical and should be evaluated further in future studies. If confirmed, it would likely be species- and metabolite-specific, rather than universal.

Just as depsidones were the main components of the G-DEN specimens, they were probably also the most abundant constituents in the three extracts of *L. occultum* (L-OCU1, L-OCU2 and L-OCU3); furthermore, this species belongs to the same family, Graphidaceae, as G-DEN. The finding (via LC–MS analysis) that norstictic acid (**9**) was tentatively the major metabolite in all *L. occultum* extracts was consistent with the reports by Mangold et al. [80] and Rivas Plata et al. [81]; however, these authors also indicated that stictic acid (**6**) was another relevant depsidone for the species. Nonetheless, while L-OCU1 and L-OCU3 (which contain (**6**) as the second most likely component) were consistent with the scientific reports, L-OCU2 was not; the L-OCU2 extract exhibited a different chemical profile (more components (20), many of which were unknown). This difference in the composition of the three specimens (collected in different locations and at different times of the year: L-OCU1 (Tubará)—July 2021, dry season; L-OCU2 (Piojó)—December 2021, dry season; L-OCU3 (Luruaco)—October 2023, rainy season) may be hypothetically related to the influence of ecological factors (greater/lesser canopy cover), edaphic/seasonal factors and geographic distribution, which could affect the production of secondary metabolites by these organisms [82]. This apparent trend was similar to that described by España-Puccini et al. [27] regarding *G. dendrogramma* collected at two different locations in the TDFs of the Department of Atlántico (northern Colombia). Therefore, the secondary metabolites produced by L-OCU3 (with the highest content of norstictic acid—82%) could suggest distinctive adaptations due to greater water availability and/or possible biotic interactions [82,83]; although this interpretation is speculative, it will require future research for verification.

As for the content of lichen substances in the H-LEP extract, the UV absorption data (Figure S4F) for the most abundant metabolite, presumptively identified as schizopeltic acid (**10**), were consistent with those data published in the reviewed scientific literature, i.e., UV absorption bands at 241 nm, 274 nm, 290 nm, 301 nm, and 314 nm, according to Santesson [84] and Huneck et al. [85]. In addition, the UV spectra data (Figure S4F,G) for both dibenzofurans [(**10**) and 3-O-demethylschizopeltic acid (**11**)] were equivalent (indicating structural similarity), with differences only in the intensity of the absorption band at 202 nm and the shift of the band between 214 nm and 219 nm, which was greater in (**10**) than in (**11**), a difference that could be attributed to the absence/presence of the CH_3_ group in their structures. This spectroscopic phenomenon has also been observed in other dibenzofurans, depsides, and depsidones (with/without a CH_3_ group), e.g., ascomatic/norascomatic acids, isopatagonic/2-O-methylisopatagonic acids, and norstictic/stictic acids [27,86].

Nonetheless, mass spectrometry analysis (ESI–MS, MS/MS and DI–MS/MS) was essential for the structural recognition of these constituents [87]; for example, the mass spectra of schizopeltic and 3-O-demethylschizopeltic acids in the positive and negative modes showed protonated and deprotonated molecular ions ([M+H]^+^/[M − H]^−^) with *m*/*z* 359.0586/357.0252 and *m*/*z* 345.0325/343.0070, one to one. From these molecular ions, the hypothetical fragmentation pattern was similar for both presumed dibenzofurans, with a difference of 14 mass units (i.e., equivalent to a CH_3_ group) when comparing each fragment ion of one compound with that of the other compound (Figure S5F,G). Moreover, the typical fragmentation in the positive mode corresponded to the loss of H_2_O and CO_2_ molecules from [M + H]^+^, while in negative mode, the most important ion was the result of the decarboxylation process from [M − H]^−^. Due to the limited fragmentation of this type of structure in ESI–MS analysis (in both modes), the two possible constituents were analyzed by MS/MS (QqQ) in positive mode (Figure S6).

**Scheme 3:**
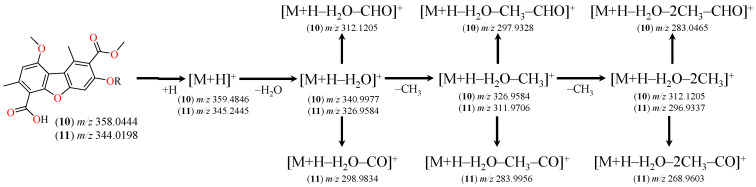
Suggested fragmentation route for dibenzofurans analyzed by MS/MS.

Thus, the comparison of the mass spectra of the product ions from (**10**) and (**11**) revealed a high degree of similarity in terms of ion distribution and intensity, with a difference of 14 mass units (i.e., one CH_3_ group) when comparing the spectra ion by ion, as previously mentioned during the ESI–MS analysis. The likely fragmentation pathway (Scheme 3) for these dibenzofurans, based on their MS/MS spectra, is as follows: after the protonation process and generation of [M + H]^+^ ions, the product ions resulted from the sequential elimination of one H_2_O molecule and -CH_3_ groups (up to 2); in turn, some product ions were formed due to the loss of the -CHO group (in the fragmentation of schizopeltic acid) and the CO molecule (in the fragmentation of 3-O-demethylschizopeltic acid) from the product ions following the elimination of H_2_O.

The DI–MS/MS spectra (complementary analysis) of presumed schizopeltic acid (Figure S7), both in positive/negative modes, showed some similarity in ion distribution and intensity to the mass spectra of (**10**) described by Huneck et al. [85] and Sargent and Stransky [88], although these authors obtained the mass spectra using the electron ionization mode.

**Scheme 4:**
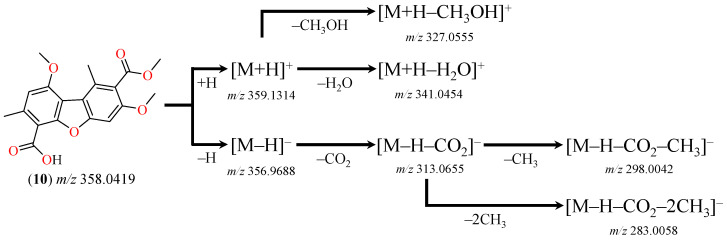
Proposed dissociative route for schizopeltic acid analyzed by DI–MS/MS.

The proposed fragmentation pathway (Scheme 4) for (**10**) via DI–MS/MS analysis is as follows: after the protonation process, the molecular ion [M + H]^+^ (100%) competitively produced fragments at *m*/*z* 341.0454 (ca. 16%) and *m*/*z* 327.0555 (ca. 16%) due to the release of H_2_O (*m*/*z* 18.0860) and CH_3_OH (*m*/*z* 32.0759), respectively. Meanwhile, in negative mode, once the deprotonated molecular ion [M − H]^−^ (ca. 6%) was formed, a CO_2_ (*m*/*z* 43.9033) molecule was released and a daughter ion at *m*/z 313.0655 (base peak) was generated; then, from this decarboxylated fragment, by two different routes, two daughter ions were produced at *m*/*z* 298.0042 (95%) and *m*/*z* 283.0058 (ca. 11%) as a result of the scission of the CH_3_ (*m*/*z* 15.0613) and 2CH_3_ (*m*/*z* 30.0597) groups, separately.

The comparison of the lichen substances (potential dibenzofuran compounds) found in the H-LEP extract in this study differed from those described in the reviewed scientific literature, i.e., Rincón-Espitia et al. [89] and Bungartz et al. [40] reported that lecanoric acid (didepside) was the main constituent of H-LEP from Juan de Acosta (Department of Atlántico) and Galapagos Island, respectively. Therefore, it is reported for the first time that the main constituents of a Colombian H-LEP specimen could be schizopeltic acid (**10**) and 3-O-demethylschizopeltic acid (**11**). It is important to note that schizopeltic acid is a dibenzofuran that is less common in lichens (restricted to certain genera—*Combea*/*Schizopelte*, *Psoroma*, *Roccella*, *Crocynea*, *Lecanactis*, *Lepraria*, *Leprocaulon* and *Leproloma*) than usnic acid (dihydrobenzofuran), which is the most common dibenzofuran of lichens [30,90] and (**10**) has been isolated/detected as a primary/secondary component in *Schizopelte californica*, *Reinkella parishii*, *Combea mollusca*, *Rocella hypomecha*, *R. capensis*, *Gyrographa gyrocarpa*, *Lecanactis abietina*, *Schismatomma umbrinum*, *L. subfarinosa*, *L. totarae*, *Sagenidium citrinum*, and *Lecidea flavothallia*, etc. [30,48,55,84,91,92,93,94,95], but not in the genus *Helminthocarpon* (Arthoniaceae, Arthoniales) [96].

Similarly, 3-O-demethylschizopeltic acid has been restricted in lichens and has only been isolated from *R. hypomecha* (Roccellaceae, Arthoniales) [92]. These findings raise a fundamental question why this Colombian H-LEP specimen produced these atypical secondary metabolites. One speculative hypothesis is that the occurrence of these dibenzofurans in phylogenetically distant lineages (Roccellaceae and Arthoniaceae) within the order Arthoniales could be considered potential evidence of convergent evolution or the conservation of shared ancestral biosynthetic pathways, a pattern consistent with the convergent nature of lichenization itself [86,97,98]. Nonetheless, this “hypothesis” would need to be demonstrated (future research directions) through phylogenetic reconstruction, analysis of biosynthetic gene cluster, reconstruction of ancestral states, or comparative genomic evidence.

Finally, as mentioned earlier, the main metabolites of a Colombian P-OCH specimen were most likely anthraquinone derivatives (including chlorinated anthraquinones), as well as a xanthone-like metabolite. Henceforward, the UV–Vis spectra amongst anthraquinones (both non-halogenated and chlorinated) were similar, showing characteristic absorption bands (π,π* and n,π* transitions) for the compound type at 224–226 nm, 269–274 nm, 288–291 nm, 308–312 nm, and 434–438 nm, which coincided with those reported by Navas Diaz [99] and Cohen and Tower [100]. On the other hand, the UV–Vis spectrum of the most abundant compound (unknown) in P-OCH showed absorption bands at 254 nm, 265 nm, 305 nm and 366 nm, which were similar to the characteristic bands of xanthones produced by the mycobionts of *Pyrenula japonica* and *P. pseudobufonia*, according to Tanahashi et al. [101], as well as those of other lichens [102] and other plant xanthones [103].

With regard to the mass spectrometry analysis of these lichen substances, it was observed that they differed from one another, i.e., the mass spectra of the seven chlorinated anthraquinones were easily distinguished by their isotopic ions (separated by two mass units) due to the ^34.969^Cl and ^36.966^Cl atoms and their ratio (3:1); consequently, the base peak ions for all chlorinated anthraquinones, in both positive and negative modes, were their protonated and deprotonated molecular ions ([M + H]^+^/[M − H]^−^), just as for the non-halogenated anthraquinones. It is worth noting that all the mass spectra of the possible anthraquinones were poor in fragment ions, since they are highly stable polycyclic aromatic molecules (anthracene derivatives); under ESI conditions (10–20 eV), these molecules can only be ionized by producing mainly molecular ions [104,105], for which all mass values are recorded in Table S2. Consequently, due to the lack of fragmentation for this type of structure, only the two presumed major constituents (7-chloroemodin and unidentified xanthone derivative) were analyzed by MS/MS and DI–MS/MS, respectively.

**Scheme 5:**
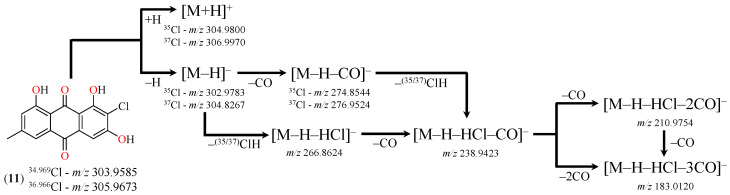
Suggested MS/MS fragmentation route for 7-chloroemodin.

Then, MS/MS analysis (Figure S6) in negative mode produced, following deprotonation of (**12**), the corresponding molecular ions [M − H]^−^ associated with the atoms ^34.969^Cl and ^36.966^Cl, as well as the daughter ions of both [M − H]^−^ (Supplementary Materials) to confirm that [M − H]^–^ at *m*/*z* 304.8267 was the ^36.966^Cl-isotopic molecular ion and not another compound. Consequently, the most probable product ions of the parent ions were typical for the non-halogenated structures of anthraquinones, i.e., successive losses of CO molecules (*m*/*z* 27.9669; up to five molecules—Scheme 5), similar to that reported by Zeng et al. [106], generating fragments at *m*/*z* 274.8544 (*m*/*z* 276.9524—^36.966^Cl), *m*/*z* 238.9423 (51%), *m*/*z* 210.9754 (100%), *m*/*z* 183.0120 (9%), and *m*/*z* 154.9522. A specific daughter ion at *m*/*z* 266.8624 resulted from the ejection of an HCl molecule (*m*/*z* 36.1159/*m*/*z* 37.9643) from both [M − H] ^–^, observed in each MS/MS spectrum (Figure S6).

**Scheme 6:**
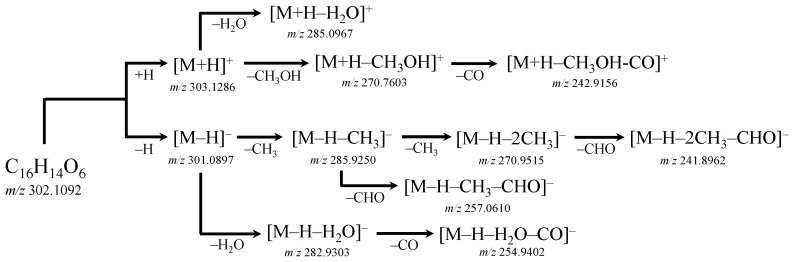
Suggested cleavage pathway for an unknown xanthone analyzed by DI–MS/MS.

Finally, the mass spectra (positive/negative modes) of the unidentified xanthone-type metabolite showed [M + H]^+^/[M − H]^−^ as the base peak ions in the current ESI–MS analysis; in addition, the DI–MS/MS analysis (Figure S8) in positive/negative modes again produced [M + H]^+^/[M − H]^−^ as the base peaks, with *m*/*z* 303.1208/*m*/*z* 301.0897, from which product ions were formed (Scheme 6); i.e., in positive mode, four daughter ions—at *m*/*z* 285.0967, *m*/*z* 270.7603, *m*/*z* 242.9156, and *m*/*z* 212.8406—which resulted from the elimination of molecules of H_2_O (*m*/*z* 18.0241), CH_3_OH (*m*/*z* 32.3605), CO (*m*/*z* 27.8447), and CH_2_O (*m*/*z* 30.0750); in negative mode, six daughter ions were formed—at *m*/*z* 285.9250, *m*/*z* 282.9303, *m*/*z* 270.9515, *m*/*z* 257.0610, *m*/*z* 254.9402 and *m*/*z* 241.8962—resulting from the removal of groups/molecules of -CH_3_ (*m*/*z* 15.1647), H_2_O (*m*/*z* 18.1594), 2 (-CH_3_) (*m*/*z* 30.1382), -CHO (*m*/*z* 29.0553), and CO (*m*/*z* 27.9901).

The negative-mode MS/MS spectrum of this unknown compound showed similarity in both ion distribution and intensity (with a difference of one in mass unit, 1 *m*/*z*) to the mass spectrum (by EI mode) of a tetraoxygenated xanthone (C_16_H_14_O_6_, M_W_: 302.0793 u, λ_UV/vis_: 252 nm, 275 nm, 300 nm and 370 nm) isolated from *Centaurium littorale* and chemically characterized by Van der Sluis and Labadie [107]. Moreover, when the literature review was broadened to include fungal extrolites, it was found that *Seimatosporium* sp. (pestalotioid fungus) produced a tetraoxygenated xanthone (seimatoxanthone A, λ_UV/vis_: 253 nm, 285 nm, and 350 nm), with the chemical formula C_16_H_14_O_6_ and M_W_ of 302.0775 u [108]. Thus, considering the aforementioned scientific literature and based on the analysis of UV–Vis absorption bands and MS/MS spectra, it could be suggested that the unidentified substance, in addition to presumably being a xanthone, could tentatively be of the tetraoxygenated type and would fit C_16_H_14_O_6_ ([M + 1]: 303.0135 u); however, the unidentified xanthone-like metabolite did not match any currently consulted lichen-derived xanthone. Lastly, it should be noted that the presumed chemical composition determined by LC–MS of the Colombian specimen of P-OCH would constitute the first detailed compositional study of the species.

Based on a comparison with other authors, it was found that, according to Miranda-González et al. [109], anthraquinones are biomarkers for species of *P. ochraceoflava* complex [species from Brazil (7-chloroemodin/emodin), Cuba, E.E.U.U., Mexico (parietin), Cook Island (7-chloroemodin) and Galapos Island (fragilin traces)], which was consistent with this research (7-chloroemodin/emodin/parietin). However, the main lichen substance (a xanthone-type metabolite) for the Colombian species was not found in other species of the complex, which were only analyzed by TLC. Therefore, the variability of anthraquinones in chemical composition (chemotypes) within the *P. ochraceoflava* complex could hypothetically be due to both evolutionary differences and environmental conditions, as suggested by Miranda-González et al. In addition, anthraquinones/xanthones are biomarkers in other species of *Pyrenula*, e.g., *P. awasthii* sp. nov. (unknown anthraquinone), *P. spissitunicata* (lichexanthone), *P. endocrocea* (orange anthraquinone), *P. hawaiiensis* (lichexanthone), *P. ochraceoflavens* (anthraquinones), *P. ochraceoflava* var. *pacifica* (anthraquinones), etc. [110,111,112,113].

Finally, the hierarchical tree plot showed that the studied lichens from the TDF remnants of the Department of Atlántico contained, tentatively, secondary metabolites of the typical substances of lichens. Even though the Department of Atlántico is a small department in Colombia, its TDF is one of the most fragmented and has a high/permanent loss of tree cover due to anthropogenic activities [114]. This probable chemodiversity in these lichens was presumptively represented by molecules, such as: 2′-O-methylperlatolic acid, ovoic acid, gyrophoric acid, norstictic acid, stictic acid and protocetraric acid, along with schizopeltic acid, 7-chloroemodin and emodin (and a xanthone-like metabolite), which are characteristic lichen substances related to evolutionary patterns for these types of organism or eco-evolutionary drivers of global lichen distribution, as stated by Boustie and Grube [115], Allen and Lendemer [116] and Schweiger et al. [117]. Of course, there are some exceptions, e.g., *H. leprevostii* specimen; it is still not clear why it would produce those secondary metabolites (schizopeltic acid and its derivative).

It should be noted that, unfortunately, it was not possible to isolate the putatively identified secondary metabolites from the lichen species due to their limited biomass. Nonetheless, the next stages of this research aim to isolate the mycobionts from the lichens and provide them with appropriate/optimal conditions for their growth, development, and metabolite production, through biotechnological processes.

## 5. Conclusions

This study expands knowledge about the potential content of secondary metabolites, presumably found in corticolous crustose lichens from Colombian TDFs, by establishing distinctive chemical profiles for all specimens studied. Some of these presumptive constituents could coincide with those from the existing scientific literature, while others did not, and are mentioned/reported here for the first time for certain lichen specimens (e.g., potentially undescribed *Cryptothecia* sp. and *Helmithocarpon leprevostii*). Furthermore, the likely compositional discrepancy observed (based on the types of constituents and their relative amounts) between some of these chemical profiles could hypothetically be attributed to geographical, seasonal, or ecological factors. Finally, the supposed chemical diversity of 10 of the 11 analyzed lichen specimens from some TDF remnants in the Department of Atlántico could likely be reflected in the common types of secondary metabolites tentatively identified, e.g., depsidones, depsides, dibenzofurans, and anthraquinones/xanthones, despite (i) the small area of this department and (ii) the highly fragmented nature of its TDF.

## Figures and Tables

**Table 1:** Taxonomical information of the 11 lichen specimens under study.

Acronym	Lichen Species	Family	Voucher
C-SP1	*Cryptothecia* sp.	Arthoniaceae	CM3094b
C-SP2	*Cryptothecia* sp.	Arthoniaceae	CM3017
C-SCR	*Cryptothecia scripta* G. Thor	Arthoniaceae	CM3616
G-DEN1	*Graphis dendrogramma* Nyl.	Graphidaceae	CM3453
G-DEN2	*Graphis dendrogramma* Nyl.	Graphidaceae	CM3652
L-OCU1	*Leucodecton occultum* (Eschw.) Frisch	Graphidaceae	CM3445
L-OCU2	*Leucodecton occultum* (Eschw.) Frisch	Graphidaceae	CM3484
L-OCU3	*Leucodecton occultum* (Eschw.) Frisch	Graphidaceae	CM3661
H-LEP	*Helminthocarpon leprevostii* Fée	Arthoniaceae	CM3465
P-OCH	*Pyrenula ochraceoflava* (Nyl.) R.C. Harris	Pyrenulaceae	CM3594
A-SEM	*Allographa seminuda* (Müll. Arg.) Lücking & Kalb	Graphidaceae	CM3010

**Table 2:** Mass analysis [LC–MS (ESI–Iontrap, positive/negative modes)] of the two main constituents (≥50–99%) tentatively identified in the total extracts of the 10 lichen specimens.

Lichen	Tentative Identification	Chemical Formula	Identification Methods	[M + H]	[M − H]	Adduct/Fragment Ions		MW_Exp_ (u)
						+Mode	−Mode	
C-SP1 C-SP2	2′-O-Methylperlatolic acid (**1**)	C_26_H_34_O_7_	R_f_ (TLC), UV–Vis, HRMS, MS/MS	459.1682	457.2025	939.4577, 441.2030 221.0572	915.4964, 237.0472, 193.0442	458.1637
	Isohyperplanaic acid (**2**)	C_29_H_40_O_7_	UV–Vis, HRMS, MS/MS	501.2006	499.2633	1023.4913, 523.2936	1021.5699	500.2319
C-SCR	Gyrophoric acid (**4**)	C_24_H_20_O_10_	R_f_ (TLC), R_t_ (LC), UV–Vis, HRMS, fragmentation	469.1312	466.9358	959.1413, 300.9787, 150.9288	957.1002, 316.8664	468.0335
	Ovoic acid (**5**)	C_25_H_22_O_10_	R_t_ (LC), UV–Vis, HRMS, fragmentation	483.0482	480.9406	987.1879, 314.9926, 164.9519, 150.9322	985.1082, 330.8608	481.9944
G-DEN1 G-DEN2 L-OCU1 L-OCU3	Stictic acid (**6**)	C_19_H_14_O_9_	R_f_ (TLC), UV–Vis, HRMS, MS/MS, comparison compound prev. isolated	387.0487	385.0265	795.1106, 369.0433	771.1410, 793.1818	386.0376
G-DEN1	Constictic acid (**7**)	C_19_H_14_O_10_	R_f_ (TLC), UV–Vis, HRMS, MS/MS	403.1250	401.0316	827.1188, 769.1063	803.1182, 825.1250	402.0783
G-DEN2 L-OCU1 L-OCU2 L-OCU3	Norstictic acid (**9**)	C_18_H_12_O_9_	R_f_ (TLC), UV–Vis, HRMS, MS/MS	373.0436	370.9912	767.0357, 395.0410	743.0995, 327.0119	372.0174
H-LEP	Schizopeltic acid (**10**)	C_19_H_18_O_7_	R_f_ (TLC), UV–Vis, HRMS, MS/MS, DI–MS/MS	359.0586	357.0252	739.0630, 381.0513 341.0461	715.1623, 313.0149	358.0419
	3-O-Demethylschizopeltic acid (**11**)	C_18_H_16_O_7_	UV–Vis, HRMS, MS/MS,	345.0325	343.0070	711.0412, 367.0000, 327.03576	687.1043 298.9887	344.0197
P-OCH	7-Chloroemodin (**12**)	C_15_H_9_ClO_5_	R_f_ (TLC), UV–Vis, HRMS, MS/MS	304.9756 307.0000	302.9412 304.8750	631.0000, 419.2783	629.0583, 957.0466	303.9584 305.9375
	Unknown xanthone-type metabolite	C_16_H_14_O_6_	R_f_ (TLC), UV–Vis, HRMS, MS/MS, DI–MS/MS	302.9630	300.9250	511.9864, 627.0100	602.9844, 625.0000	301.9440

MW: Molecular weight; Exp: Experimental; all presumptively assigned chemical names were compared with those available in the scientific literature and databases.

## Data Availability

All data from this research are included in the Supplementary Materials.

## Supplementary Materials

The following supporting information can be downloaded at: https://www.mdpi.com/article/10.3390/jof12070526/s1, Table S1. Names, voucher numbers (ONU herbarium) and collected locations of the species selected and identified for this study; Figure S1. Thallus of *Pyrenula ochraceoflava* (CM3594) removed with a ROLSON Quality Tools 7” classic mini steel-wire brush; Figure S2. Chromatograms (R_t_ window: 3–20 min) obtained by LC–DAD/MS (190–600 nm) of the total extracts of the lichens; Figure S2 continued. Chromatograms (R_t_ window: 3–20 min) obtained by LC–DAD/MS (190–600 nm) of the total extracts of the lichens; Figure S3. Thin-layer chromatography (TLC) profiles of four lichen samples analyzed: A. C-SP2 (CM3017), B. G-DEN2 (CM4652), C. L-OCU (CM3661), and D.C-SCR (CM3616).; Figure S4. UV spectra obtained by LC–DAD/MS (190–600 nm) of the most abundant compounds in lichens; Figure S4 continued. UV spectra obtained by LC–DAD/MS (190–600 nm) of the most abundant compounds in lichens; Figure S5. Mass spectra obtained by ESI–iontrap–MS (positive and negative modes) of the most abundant compounds in lichens; Figure S5 continued. Mass spectra obtained by ESI–iontrap–MS (positive and negative modes) of the most abundant compounds in lichens; Figure S6. Mass spectra obtained by MS/MS (QqQ) (positive or negative modes) of the most abundant compounds in three lichens; Figure S7. Mass spectra obtained by DI–MS/MS (QqQ) (positive and negative modes) of schizopeltic acid (from *H. leprevostii*); Figure S8. Mass spectra obtained by DI–MS/MS (QqQ) (positive and negative modes) of an unidentified xanthone-like metabolite (from *P. ochraceoflava*); Table S2. Constituents registered and/or identified presumptively by TLC and LC–DAD/MS (ESI) in all the extracts from 11 lichen specimens.

## Abbreviations

The following abbreviations are used in this manuscript:

A-SEM	*Allographa seminuda*
BGBM	Botanischer Garten und Botanisches Museum
CA	Cluster analysis
C-SP1/C-SP2	*Cryptothecia* sp. specimen1/specimen2
C-SCR	*Cryptothecia scripta*
DI–MS/MS	Direct infusion-tandem mass spectrometry
ESI	Electrospray ionization
G-DEN1/G-DEN2	*Graphis dendrogramma* specimen1/specimen2
H-LEP	*Helminthocarpon leprevostii*
HPLC–DAD	High-performance liquid chromatography–diode array detector
HRMS	High-resolution mass spectrometry
λ	Wavelength
LC–ESI–MS	Liquid chromatography–electrospray ionization–mass spectrometry
LC–MS/MS	Liquid chromatography–tandem mass spectrometry
L-OCU1/L-OCU2/L-OCU3	*Leucodecton occultum* specimen1/specimen2/specimen3
[M + H]^+^	Protonated molecular ion
[M − H]^−^	Deprotonated molecular ion
MI	Molecular ion
PDA	Photodiode-array detector
P-OCH	*Pyrenula ochraceoflava*
R_f_	Retention factor
R_t_	Retention time
TDF	Tropical dry forest
TLC	Thin-layer chromatography
U	Unified atomic mass unit
UV–Vis	Ultraviolet/visible
