# Supplementary material for: Chemo-Diversity and Secondary Metabolite Content in Corticolous Crustose Lichens (Fam. Arthoniaceae, Graphidaceae, Pyrenulaceae) from Remnants of Colombian Tropical Dry Forests
Source: J Fungi (Basel). 2026 Jul 17;12(7):526. doi: 10.3390/jof12070526 (PMC13413370; doi:10.3390/jof12070526)
Supplement: Supplementary file 1 [file jof-12-00526-s001.zip › jof-4298172-supplementary.pdf]

Chemo-diversity and Secondary Metabolite Content in Corticolous Crustose Lichens (fam. Arthoniaceae, Graphidaceae, Pyrenulaceae) from Remnants of Colombian Tropical Dry Forests

Pierine España-Puccini [1,2], Amner Muñoz-Acevedo [1*], Natalia A. Llanos-López [3,4], Marc Stadler [3,4], Mayar L. Ganoza-Yupanqui [5], Paula S. Burgos-Zelada [5] and María C. Martínez-Habibe [1]

1 Department of Natural Sciences, Universidad del Norte, Puerto Colombia, Colombia; amnerm@uninorte.edu.co, mhabibe@uninorte.edu.co
2 IMB – Research Group in Medicine and Biotechnology, Universidad Libre, Puerto Colombia, Colombia; pierine.espanap@unilibre.edu.co
3 Department of Microbial Drugs, Helmholtz Centre for Infection Research (HZI), Braunschweig, Germany.
4 Institute of Microbiology, Technische Universität Braunschweig, Braunschweig, Germany; natalia.llanos-lopez@helmholtz-hzi.de, marc.stadler@helmholtz-hzi.de
2 Medicinal Plant Quality Control Research Group, Faculty of Pharmacy and Biochemistry, Universidad Nacional de Trujillo, Trujillo, Perú; mganoza@unitru.edu.pe; pburgosz@unitru.edu.pe
* Correspondence: amnerm@uninorte.edu.co

Table S1. Names, voucher numbers (ONU herbarium) and collected locations of the species selected and identified for this study.

Acronym	Lichen Species	Voucher	Location	Lat. (N)	Lon. (W)	Elevation (m)
C-SP1	*Cryptothecia* sp.	CM3094b	Usiacurí	10°44'59.5"	-75°01'56.0"	141
C-SP2	*Cryptothecia* sp.	CM3017	Usiacurí	10°45'02.7"	-75°01'53.6"	135
C-SCR	*Cryptothecia scripta* G. Thor 1997	CM3616	Luruaco	10°38'03.0"	-75°12'47.9"	33
G-DEN1	*Graphis dendrogramma* Nyl.	CM3453	El Morro-Tubará	10°56'36.2"	-74°59'45.0"	211
G-DEN2	*Graphis dendrogramma* Nyl.	CM3652	Luruaco	10°38'36.0"	-75°12'40.4"	21
L-OCU1	*Leucodecton occultum* (Eschw.) Frisch	CM3445	El Morro-Tubará	10°56'41.7"	-74°59'44.0"	218
L-OCU2	*Leucodecton occultum* (Eschw.) Frisch	CM3484	Piojó	10°44'52.6"	-75°05'54.3"	250
L-OCU3	*Leucodecton occultum* (Eschw.) Frisch	CM3661	Luruaco	10°38'31.0"	-75°12'37.3"	37
H-LEP	*Helminthocarpon leprevostii* Fée	CM3465	Piojó	10°44'13.9"	-75°06'27.3"	432
P-OCH	*Pyrenula ochraceoflava* (Nyl.) R.C. Harris	CM3594	Usiacurí	10°45'01.8"	-75°01'57.9"	184
A-SEM	*Allographa seminuda* (Müll. Arg.) Lücking & Kalb	CM3010	Usiacurí	10°44'57.2"	-75°01'31.5"	113

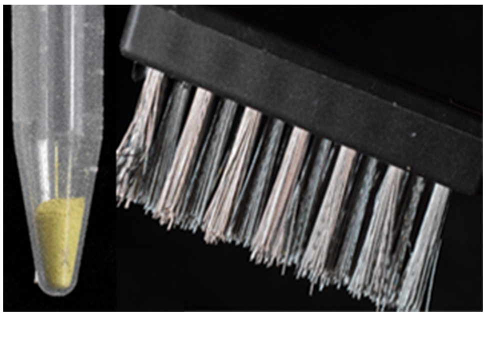

Figure S1. Thallus of *Pyrenula ochraceoflava* (CM3594) removed with a ROLSON Quality Tools 7" classic mini steel-wire brush.

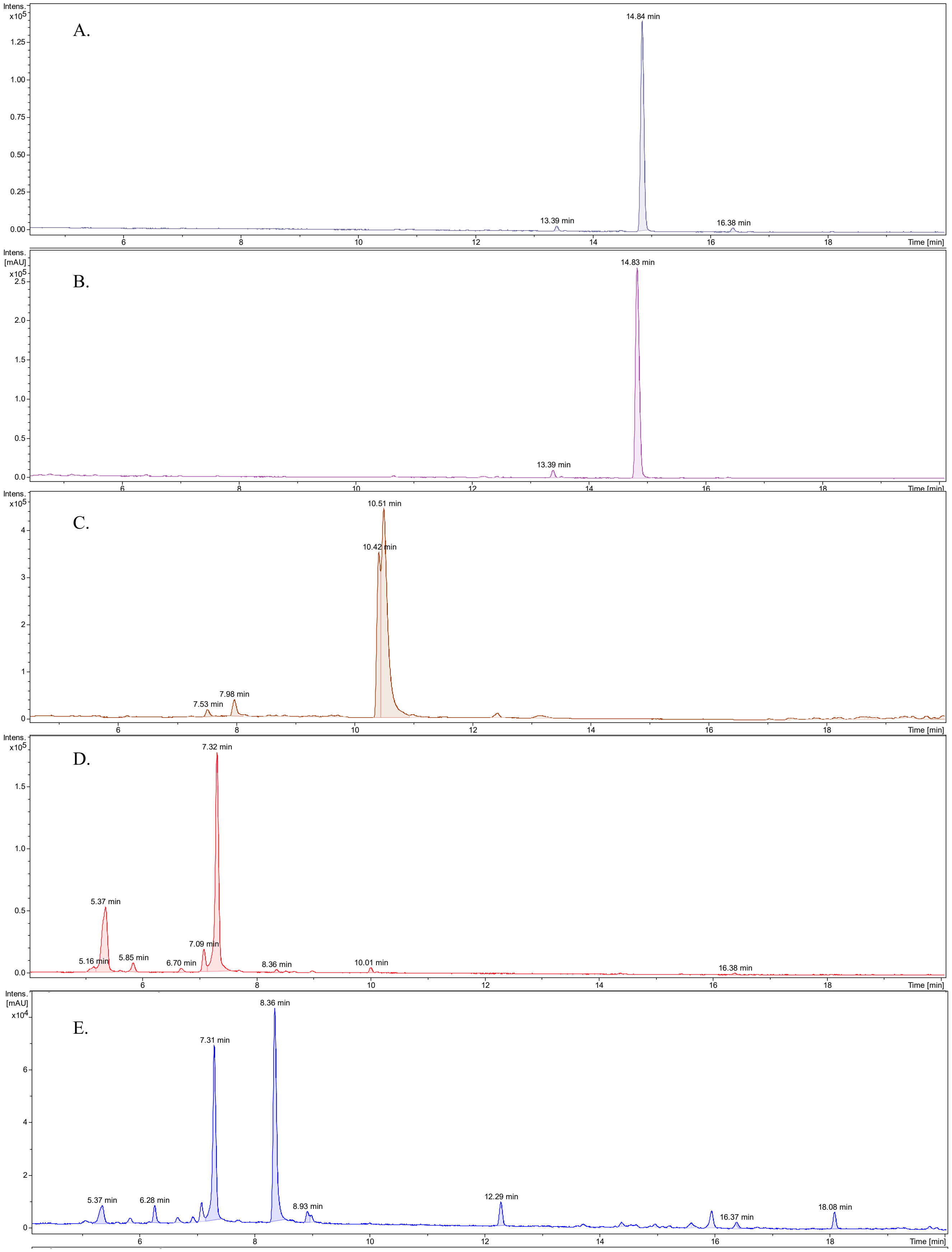

Figure S2. Chromatograms (R_t window: 3-20 min) obtained by LC-DAD/MS (190-600 nm) of the total extracts of the lichens: **A**. CM3094b (*Cryptothecia* sp. nov. - Usiacurí); **B.** CM3017 (*Cryptothecia* sp. nov - Usiacurí); **C.** CM3616 (*Cryptothecia scripta*); **D.** CM3453 (*Graphis dendrogramma* - Tubará); and, **E.** CM3652 (*G. dendrogramma* - Luruaco).

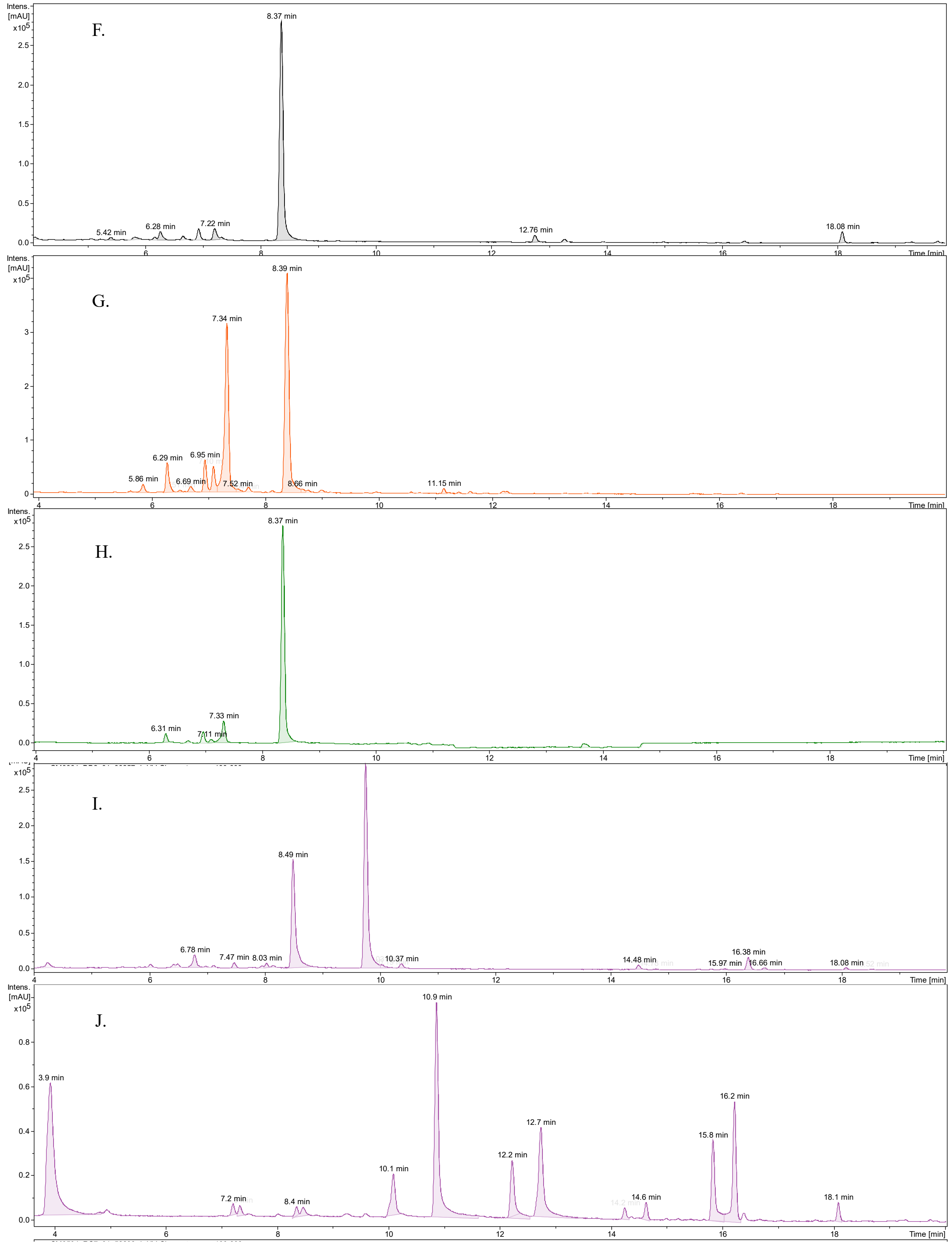

Fig. S2 continued. Chromatograms (Rt window: 3-20 min) obtained by LC-DAD/MS (190-600 nm) of the total extracts of the lichens: **F.** CM3545 (*Leucodecton occultum* - Tubará); **G.** CM3484 (*L. occultum* - Piojó); **H.** CM3661 (*L. occultum* - Luruaco); **I.** CM3465 (*Hemilthocarpon leprevostii*); and, **J.** CM3594 (*Pyrenula ochraceoflava*).

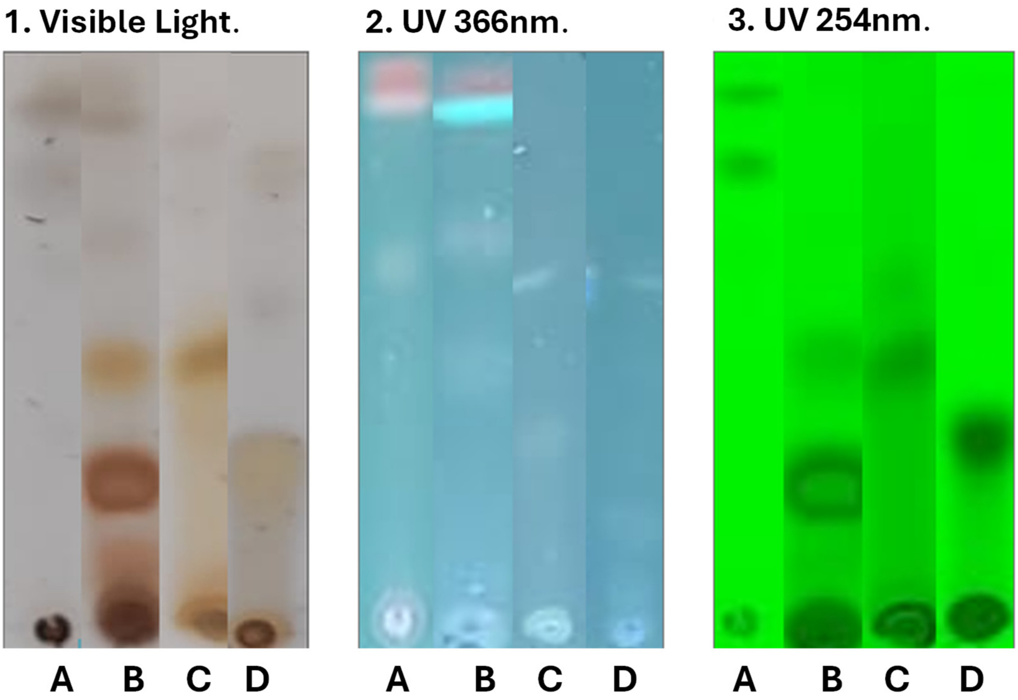

Figure S3. Thin-layer chromatography (TLC) profiles of four lichen samples analyzed: A. C-SP2 (CM3017), B. G-DEN2 (CM4652), C. L-OCU (CM3661), and D.C-SCR (CM3616).

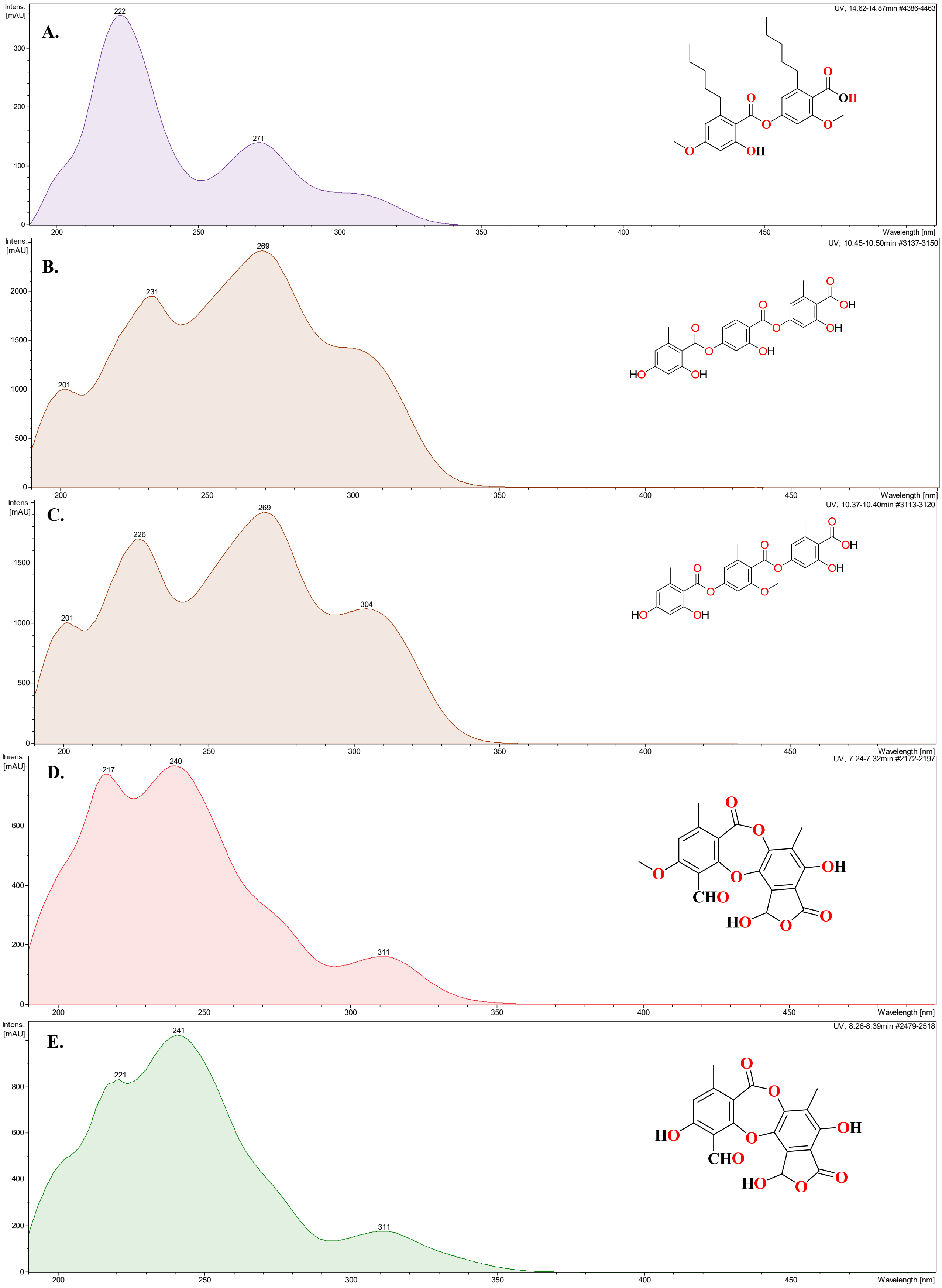

Figure S4. UV spectra obtained by LC-DAD/MS (190-600 nm) of the most abundant compounds in lichens: **A**. 2□O-Methylperlatolic acid (*Cryptothecia* spp. nov.), **B.** Ovoic acid (*C. scripta*); **C.** Gyrophoric acid (*C. scripta*); **D.** Stictic acid (*G. dendrogramma*); and, **E.** Norstictic acid (*L. occultum*) - this depsidone was the major constituents for the three *L. occultum* specimens, as well as for one of the *G. dendrogramma* specimens.

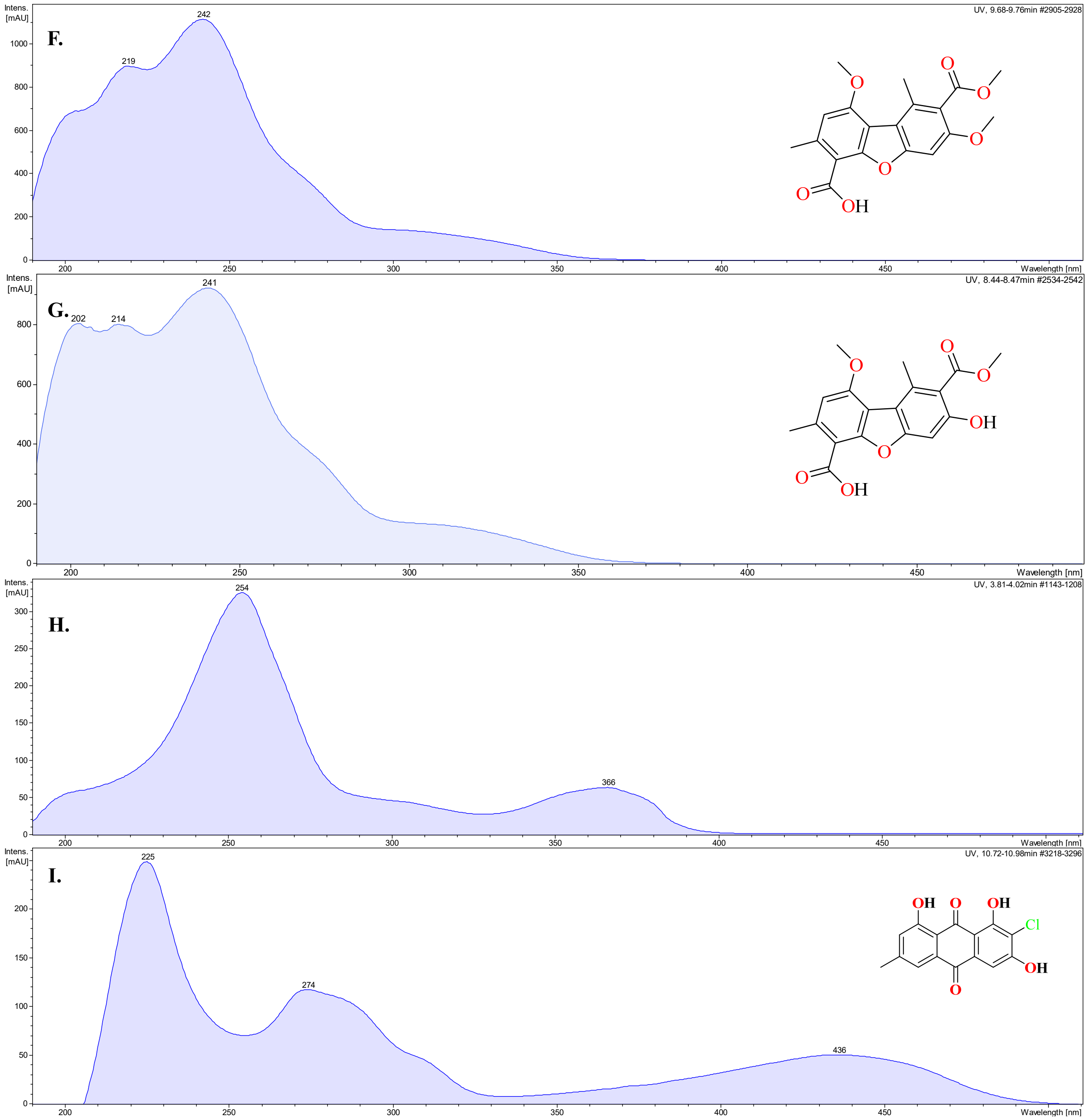

Fig. S4 continued. UV spectra obtained by LC-DAD/MS (190-600 nm) of the most abundant compounds in lichens: **F.** Schizopeltic acid (*H. leprevostii*); and, **G.** 3-O-Demethylschizopeltic acid (*H. leprevostii*); **H.** Unknown xanthone (*P. ochraceoflava*), and, **I.** 7-Chloroemodin (*P. ochraceoflava*).

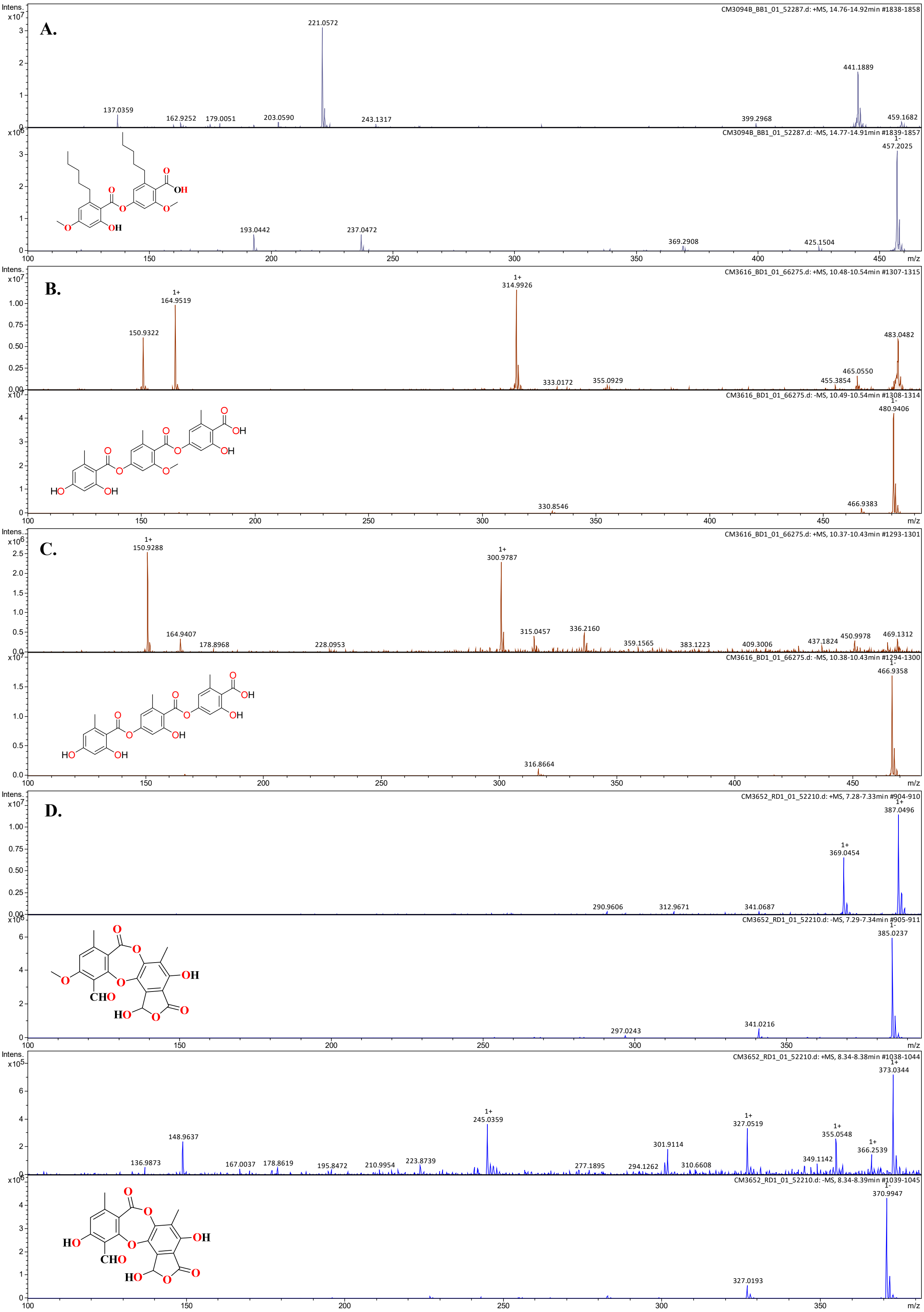

Figure S5. Mass spectra obtained by ESI-Iontrap-MS (positive and negative modes) of the most abundant compounds in lichens: **A.** 2-O-Methylperlatolic acid (*Cryptothecia* spp. nov.), **B.** Ovoic acid (*C. scripta*); **C.** Gyrophoric acid (*C. scripta*); **D.** Stictic acid (*G. dendrogramma*); and, **E.** Norstictic acid (*L. occultum*).

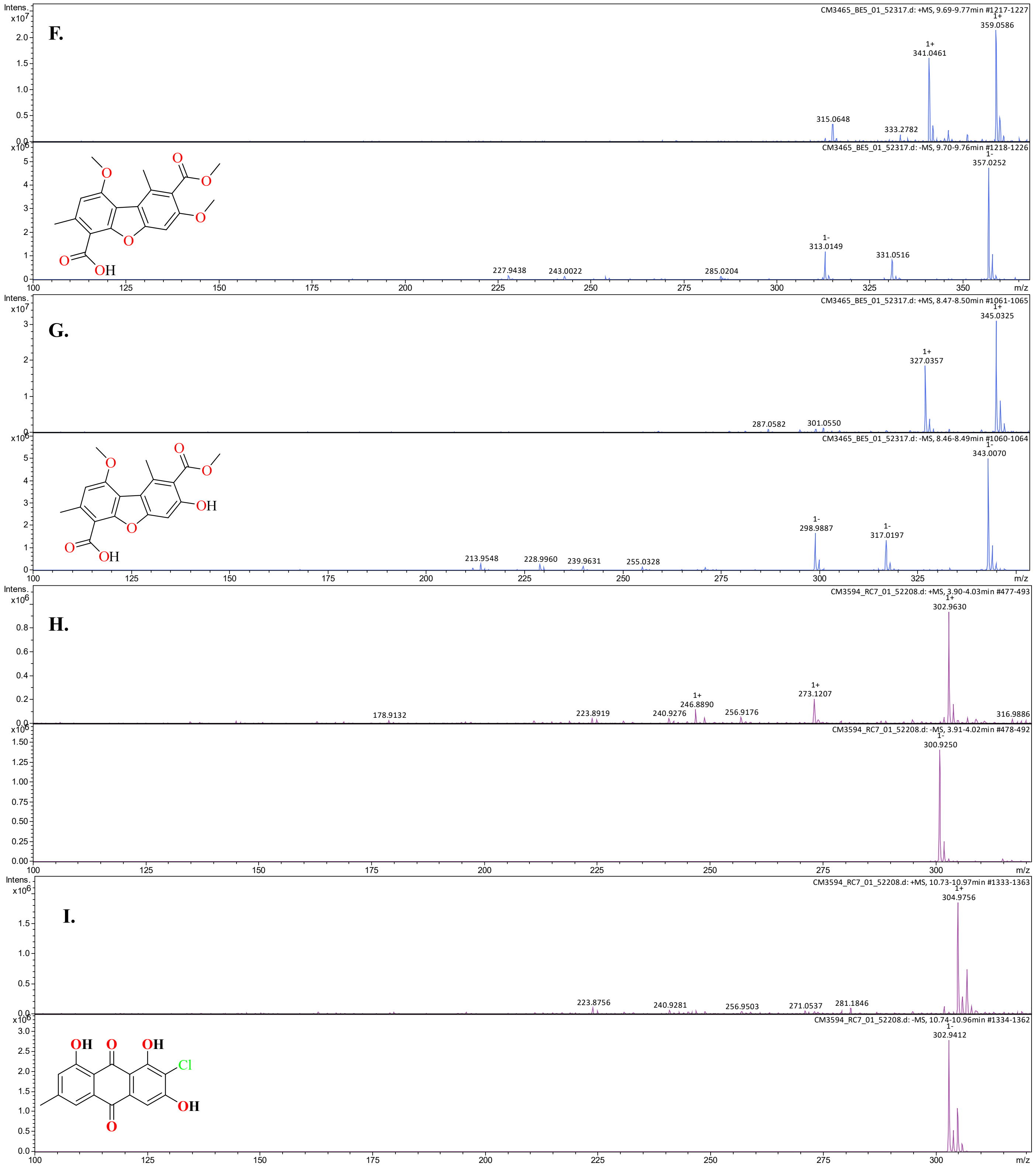

Fig. S5 continued. Mass spectra obtained by ESI-Iontrap-MS (positive and negative modes) of the most abundant compounds in lichens: **F.** Schizopeltic acid (*H. leprevostii*); and, **G.** 3-O-Demethylschizopeltic acid (*H. leprevostii*); **H.** Unknown xanthone (*P. ochraceoflava*), and, **I.** 7-Chloroemodin (*P. ochraceoflava*).

MUESTRA

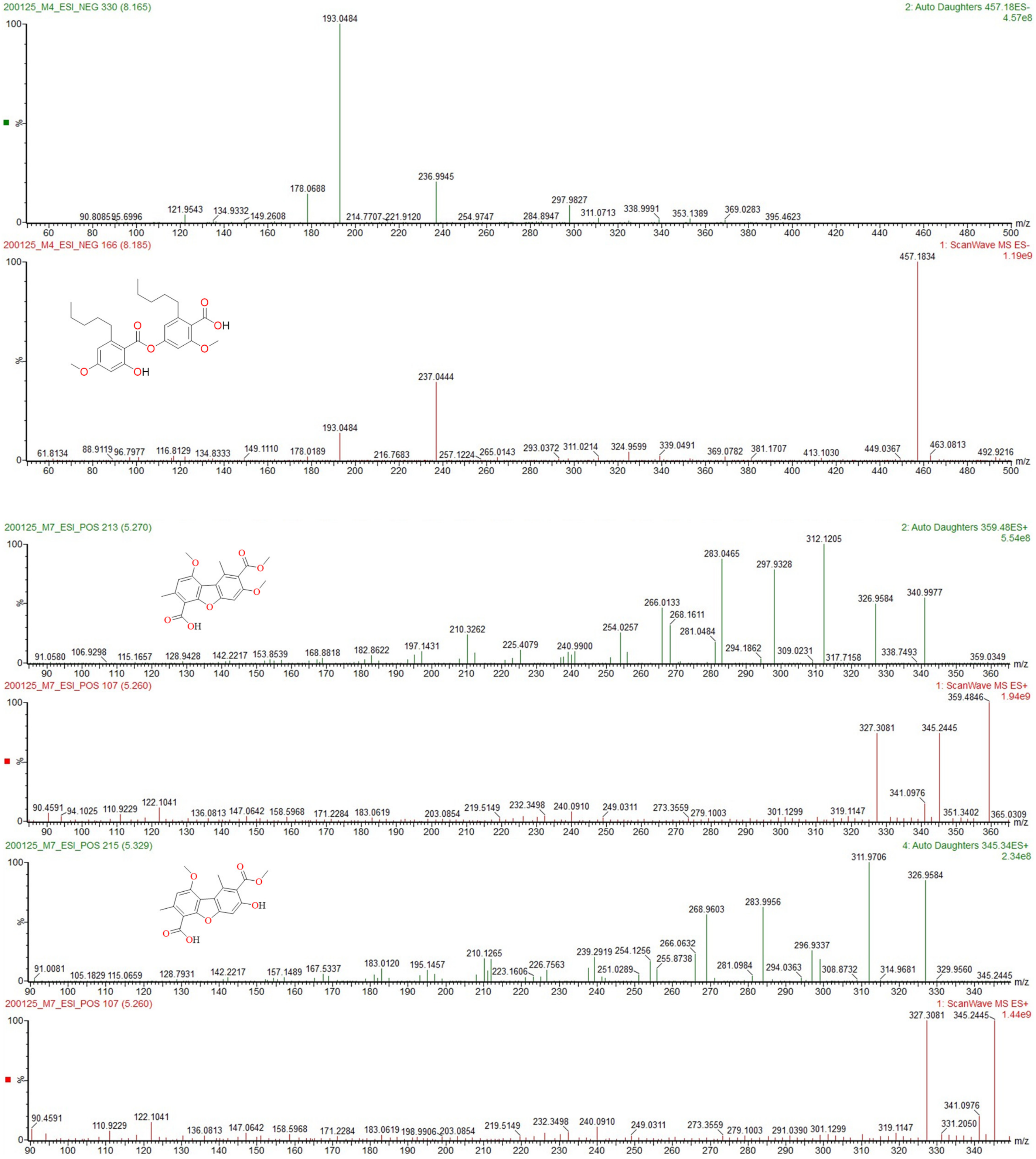

200125_M4_ESI_NEG 330 (8.165)
2: Auto Daughters 457.18ES-
4.57e8
193.0484
178.0688
236.9945
297.9827
90.8085
95.6996
121.9543
134.9332
149.2608
214.7707
221.9120
254.9747
284.8947
311.0713
338.9991
353.1389
369.0283
395.4623
200125_M4_ESI_NEG 166 (8.185)
1: ScanWave MS ES-
1.19e9
457.1834
237.0444
193.0484
61.8134
88.9119
96.7977
116.8129
134.8333
149.1110
178.0189
216.7683
257.1224
265.0143
293.0372
311.0214
324.9599
339.0491
369.0782
381.1707
413.1030
449.0367
463.0813
492.9216
200125_M7_ESI_POS 213 (5.270)
2: Auto Daughters 359.48ES+
5.54e8
312.1205
283.0465
297.9328
340.9977
326.9584
266.0133
268.1611
281.0484
254.0257
210.3262
225.4079
240.9900
197.1431
182.8622
168.8818
153.8539
142.2217
128.9428
115.1657
106.9298
91.0580
294.1862
309.0231
317.7158
338.7493
359.0349
200125_M7_ESI_POS 107 (5.260)
1: ScanWave MS ES+
1.94e9
359.4846
327.3081
345.2445
341.0976
90.4591
94.1025
110.9229
122.1041
136.0813
147.0642
158.5968
171.2284
183.0619
203.0854
219.5149
232.3498
240.0910
249.0311
273.3559
279.1003
301.1299
319.1147
351.3402
365.0309
200125_M7_ESI_POS 215 (5.329)
4: Auto Daughters 345.34ES+
2.34e8
311.9706
326.9584
268.9603
283.9956
296.9337
266.0632
255.8738
239.2919
254.1256
251.0289
210.1265
223.1606
226.7563
183.0120
195.1457
167.5337
157.1489
142.2217
128.7931
105.1829
115.0659
91.0081
281.0984
294.0363
308.8732
314.9681
329.9560
345.2445
200125_M7_ESI_POS 107 (5.260)
1: ScanWave MS ES+
1.44e9
327.3081
345.2445
341.0976
331.2050
319.1147
90.4591
110.9229
122.1041
136.0813
147.0642
158.5968
171.2284
183.0619
198.9906
203.0854
219.5149
232.3498
240.0910
249.0311
273.3559
279.1003
291.0390
301.1299
m/z
%

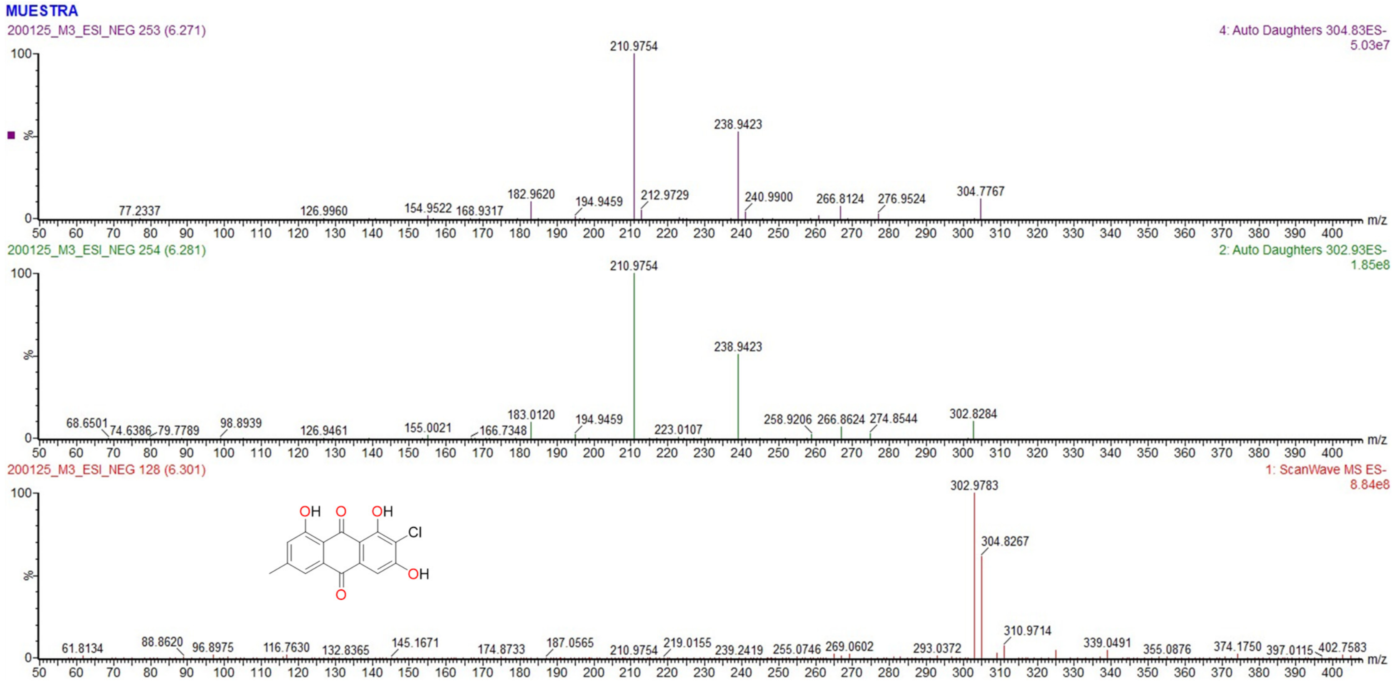

Figure S6. Mass spectra obtained by MS/MS (QqQ) (positive or negative modes) of the most abundant compounds in three lichens: 2-O-methylperlatolic acid (negative mode), schizopeltic and 3-O-demethylschizopeltic acids (positive mode) and 7-chloroemodin (negative mode).

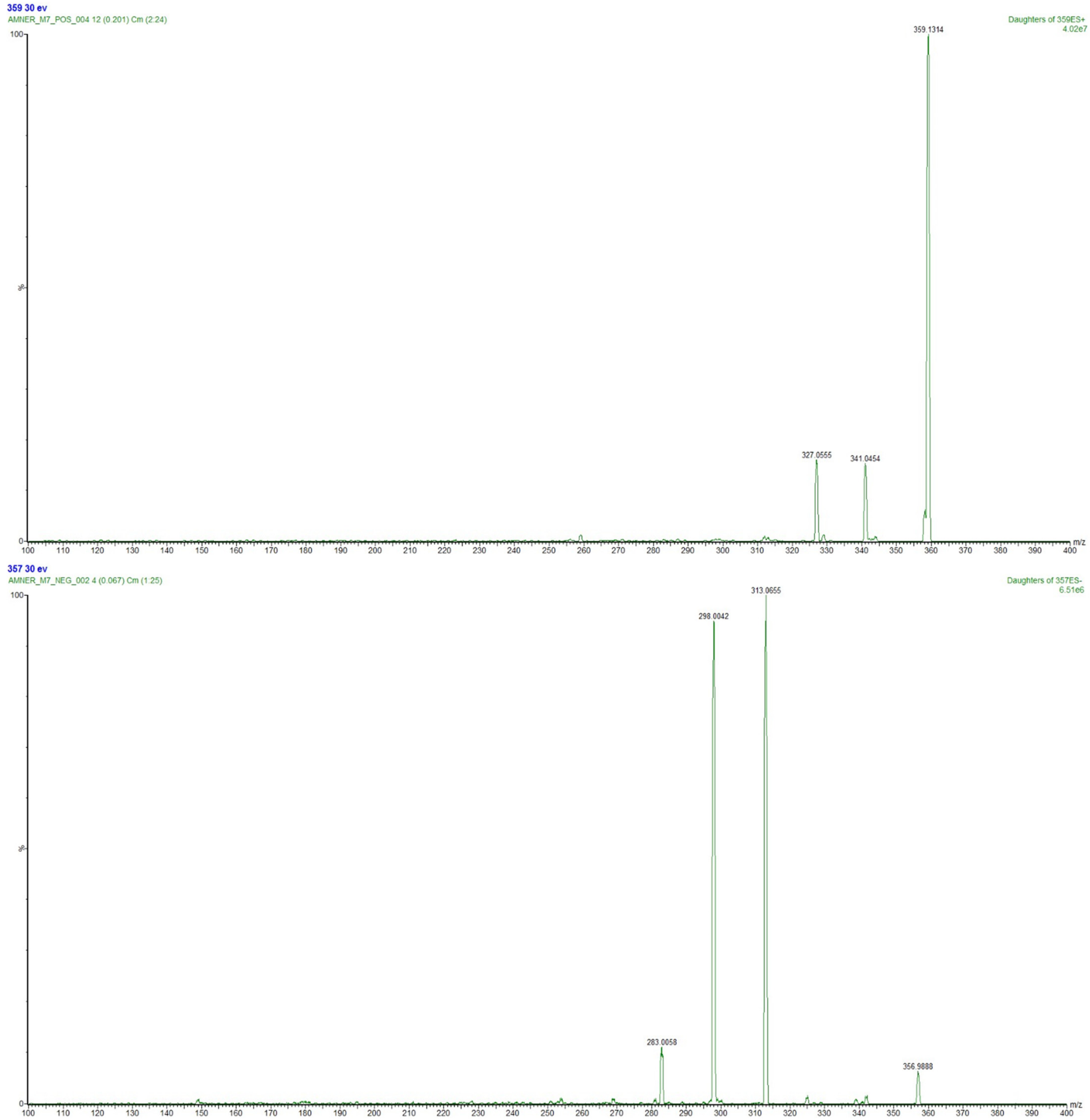

Figure S7. Mass spectra obtained by DI-MS/MS (QqQ) (positive and negative modes) of schizopeltic acid (from *H. leprevostii*).

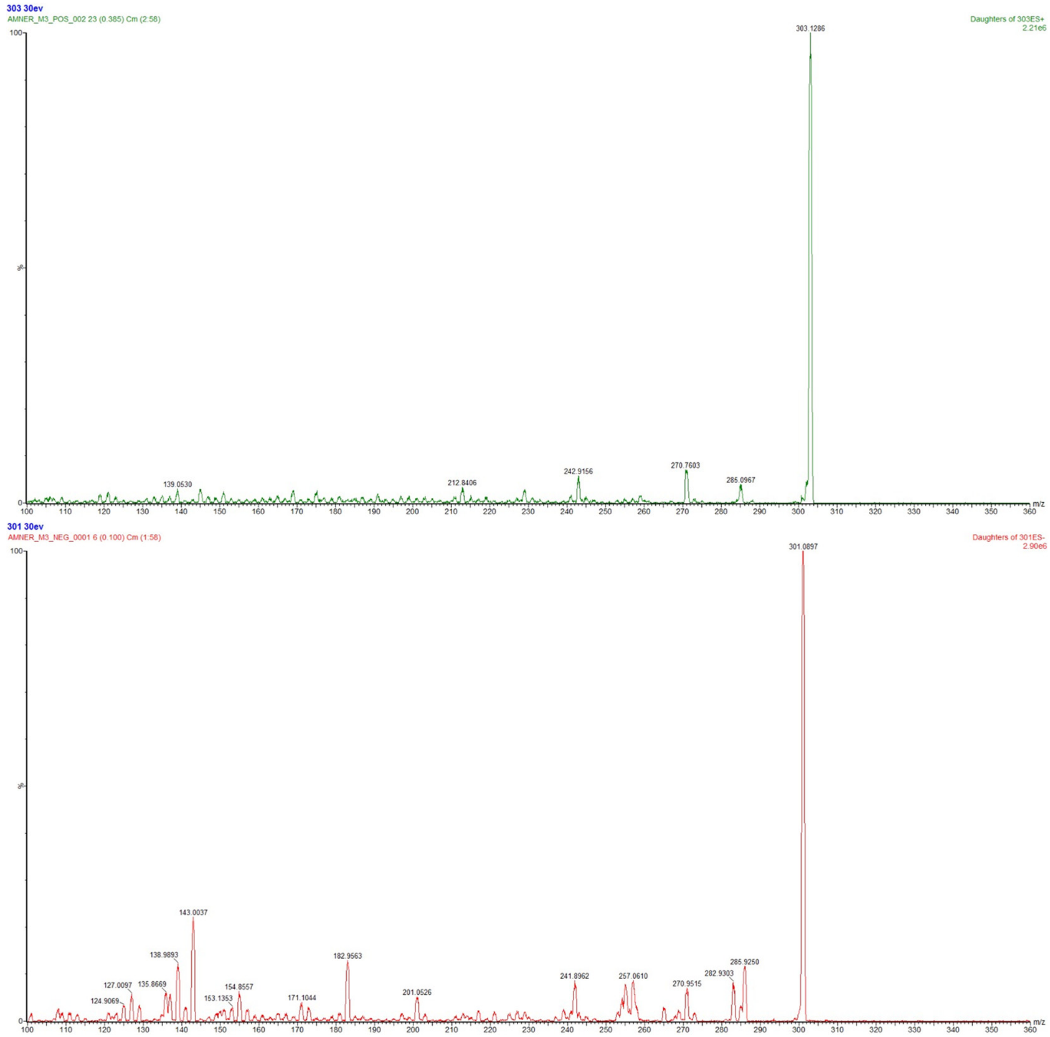

Figure S8. Mass spectra obtained by DI-MS/MS (QqQ) (positive and negative modes) of an unidentified xanthone-like metabolite (from *P. ochraceoflava*).

Table S2. Constituents registered and/or identified presumptively by TLC and LC-DAD/MS (ESI-Iontrap) in all the extracts from 11 lichen specimens

R_t, min	Tentative identification	λ_{max} (nm)	R_f values (254/366 nm)		Chemical formula	MW_{Ex} (u)	Relative amount, %										
			C_{Cl}	C_{Lt}			C-SP1	P-OCH	L-OCU1	H-LEP	G-DEN1	A-SEM	L-OCU2	G-DEN2	L-OCU3	C-SCR	C-SP2
3.94	Unknown xanthone	254	79	----	$C_{16}H_{14}O_6$	301.9440	----	26.0	tr	----	----	----	tr	----	----	----	----
4.22	Unknown compound	223	----	----	N/A	442.0888	----	----		----	----	----	0.1	----	----	----	----
4.74	Hypoconstictic acid	223	----	----	$C_{19}H_{16}O_9$	388.0314	----	----	0.1	----	----	----	tr	----	----	----	----
5.01	Unknown compound	225	----	----	N/A	352.1596	----	----		----	----	----	0.2	----	----	----	----
5.09	1,8-Dihydroxy-3,6-dimethoxyxanthone*	223	----	----	$C_{15}H_{12}O_6$	288.1720	----	----	----	----	0.5	----	----	----	----	----	----
5.16	Unknown compound	222	----	----	N/A	368.0567	----	----	----	----	1.7	----	----	----	----	----	----
5.37	Constictic acid	217	2	2	$C_{19}H_{14}O_{10}$	402.0589	----	----	tr	----	27.4	----	tr	4.8	----	----	----
5.63	Connorstictic acid	224	----	----	$C_{18}H_{14}O_9$	374.0545	----	----	0.2	----	0.3	----	----	----	----	----	----
5.86	Substictic acid	214	----	----	$C_{18}H_{12}O_9$	372.0237	----	----	1.5	----	0.2	----	0.3	0.8	----	----	----
6.28	Menegazziaic acid	218	----	----	$C_{18}H_{14}O_9$	374.0414	----	----	4.7	----	----	----	2.6	2.6	2.6	----	----
6.70	Unknown compound	222	----	----	N/A	359.9344	----	----	----	----	----	----	----	----	0.7	----	----
6.78	Unknown compound	213	----	----	N/A	488.2766	----	----	----	3.7	----	----	----	----	----	----	----
6.95	Hyposalazinic acid	224	----	----	$C_{18}H_{14}O_8$	358.0039	----	----	5.0	----	0.5	----	2.1	0.9	3.7	----	----
	Peristictic acid	224	----	----	$C_{19}H_{14}O_{10}$	402.0469	----	----	----	----	0.6	----	----	----	----	----	----
7.05	Unknown compound	234	----	----	N/A	348.1741	----	----	----	----		----	2.1	----	----	----	----
7.10	Cryptostictic acid	214	----	----	$C_{19}H_{16}O_9$	388.0838	----	----	4.2	----	5.3	----	----	3.2	1.3	----	----
7.22	Unknown compound	224	----	----	$C_{18}H_{14}O_9$	374.0446	----	0.9	----	----	----	----	----	----	----	----	----
7.34	Unknown compound	224	----	----	$C_{19}H_{16}O_8$	372.0287	----	0.7	----	----	----	----	----	----	----	----	----
7.39	Stictic acid	217	18	18	$C_{19}H_{14}O_9$	386.0351	----	----	34.8	----	59.7	----	6.6	32.9	9.1	----	----
7.47	Unknown compound	222	----	----	N/A	342.0543	----	----	----	1.1	----	----	----	----	----	----	----
7.49	Emodic acid	225	----	----	$C_{15}H_8O_7$	300.4331	----	tr	----	----	----	----	----	----	----	----	----
7.51	Unknown compound	220	----	----	N/A	331.9986	----	----	----	----	----	----	----	----	----	1.2	----
7.98	Lecanoric acid	218	----	----	$C_{16}H_{14}O_7$	317.9421	----	----	----	----	----	----	----	----	----	3.5	----
8.01	7-Chlorocitreorosein	225	----	----	$C_{15}H_9ClO_6$	318.9787<br>320.9895	----	0.3	----	----	----	----	----	----	----	----	----
8.36	Norstictic acid	225	30	30	$C_{18}H_{12}O_9$	372.0196	----	0.6	45.7	----	0.6	----	41.5	40.3	82.5	----	----
8.47	1-O-Methyl-7-chlorocitreorosein	225	----	----	$C_{16}H_{11}ClO_6$	333.9545<br>335.4289	----	0.7	----	----	----	----	----	----	----	----	----
8.49	3-O-Demethylschizopeltic acid	225	----	----	$C_{18}H_{16}O_7$	344.0194	----	----	----	31.7	----	----	----	----	----	----	----
8.93	Unknown depsidone	223	----	----	N/A	518.3132	----	----	----	----	----	----	----	1.9	----	----	----
9.02	Hypostictic acid	225	---	----	$C_{19}H_{16}O_8$	372.0606	----	----	0.4	----	0.3	----	4.4	0.9	----	----	----
9.25	1-O-Methyl-5-chloroemodin	225	----	----	$C_{16}H_{11}ClO_5$	317.9362<br>319.9800	----	0.4	----	----	----	----	----	----	----	----	----
9.59	7-Chloroteloschistin	225	----	----	$C_{16}H_{11}ClO_6$	333.9859<br>335.9892	----	0.2	----	----	----	----	----	----	----	----	----
	1-O-Methyl-7-chloroemodin	225	----	----	$C_{16}H_{11}ClO_5$	317.9383<br>319.9092	----	0.2	----	----	----	----	----	----	----	----	----
9.66	Unknown compound	201	----	----	N/A	397.9801	----	----	----	----	----	----	2.5	----	----	----	----
9.75	Schizopeltic acid	222	59	57	$C_{19}H_{18}O_7$	358.0416	----	----	----	58.4	----	----	----	----	----	----	----
10.00	Norpannarin	225	----	----	$C_{17}H_{13}ClO_6$	347.9841<br>349.9582	----	0.4	----	----	----	----	----	----	----	----	----
	Unknown compound	226	----	----	N/A	422.2098	----	----	----	----	1.0	----	----	----	----	----	----

10.06	Unknown compound	200	----	----	N/A	332.1401	----	----	----	----	----	----	5.3	----	----	----	----
10.09	Emodin	225	48	50	$C_{15}H_{10}O_5$	269.9998	----	5.6	----	----	----	----	----	----	----	----	----
10.37	Unknown compound	224	----	----	N/A	332.1617	----	----	----	1.1	----	----	----	----	----	----	----
10.42	Gyrophoric acid	221	33	33	$C_{24}H_{20}O_{10}$	468.0929	----	----	----	----	----	----	----	----	----	28.4	----
10.51	Ovoic acid	202	33	33	$C_{25}H_{22}O_{10}$	481.9957	----	----	----	----	----	----	----	----	----	66.9	----
10.87	7-Chloroemodin	225	61	59	$C_{15}H_9ClO_5$	303.9583<br>305.9375	----	23.6	----	----	----	----	----	----	----	----	----
10.97	Unknown compound	226	----	----	N/A	316.1784	----	----	----	----	----	----	1.3	----	----	----	----
11.18	Unknown compound	221	----	----	N/A	470.2531	----	----		----	----	----	2.1	----	----	----	----
11.27	Unknown compound	223	----	----	N/A	454.2492	----	----	----	----	----	----	0.8	----	----	----	----
12.19	Unknown compound	227	----	----	N/A	339.9652	----	----	0.15	----	----	----	tr	----	----	----	----
12.22	Parietin	225	74	70	$C_{16}H_{12}O_5$	284.0090	----	6.7	----	----	----	----	----	----	----	----	----
12.25	Unknown compound	227	----	----	N/A	352.0781	----	----	0.15	----	----	----	----	----	----	----	----
12.29	Nitrogen-containing compound	225	----	----	N/A	411.2884	----	----	----	----	----	----	----	4.0	----	----	----
12.69	Unknown compound		----	----	N/A	480.2930	----	----	----	----	----	----	5.7	----	----	----	----
12.74	Unknown compound type anthraquinone	226	----	----	$C_{20}H_{16}O_{13}$	464.2978	----	12.1	----	----	----	----	----	----	----	----	----
13.39	Isohyperplanaic acid	227	69	66	$C_{29}H_{40}O_7$	500.2258	1.7	----	----	----	----	----	----	----	----	----	2.6
13.94	Unknown compound	226	----	----	N/A	480.3788	----	----	----	----	----	----	1.3	----	----	----	----
14.24	Unknown compound	226	----	----	$C_{19}H_{16}O_{11}$	420.2478	----	0.9	----	----	----	----	----	----	----	----	----
14.26	Unknown compound	201	----	----	$C_{16}H_{14}O_6$	302.1881	----	----	----	----	----	----	3.9	----	----	----	----
14.41	Unknown compound	200	----	----	$C_{16}H_{14}O_6$	302.1875	----	----	----	----	----	----	10.2	----	----	----	----
14.48	Unknown compound	227	----	----	N/A	492.3467	----	----	----	1.0	----	----	----	----	----	----	----
14.62	Unknown compound	226	----	----	$C_{19}H_{16}O_{11}$	420.2626	----	1.4	----	----	----	----	----	----	----	----	----
14.84	2′-O-Methylperlatolic acid	221	63	61	$C_{26}H_{34}O_7$	458.1637	96.7	----	----	----	----	----	----	----	----	----	96.9
15.83	Unknown compound	224	----	----	N/A	448.3032	----	7.1	----	----	----	----	----	----	----	----	----
15.94	Unknown compound	227	----	----	N/A	618.4094	----	----	----	----	----	----	----	3.4	----	----	----
16.21	Unknown compound	224	----	----	N/A	448.3010	----	10.4	----	----	----	----	----	----	----	----	----
16.38	Unknown compound	227	----	----	N/A	592.2872	1.6	----	----	3.5	----	----	----	1.6	----	----	0.4
18.08	Unknown compound	228	----	----	N/A	454.1277	----	1.5	----	----	----	----	----	2.8	----	----	----
20.54	Unknown compound	200	----	----	N/A	602.4846	----	----	----	----	----	----	4.6	----	----	----	----
21.05	Unknown compound	228	----	----	N/A	398.3759	----	----	----	----	----	----	1.2	----	----	----	----

R_t: Retention time, R_f: Relative retention factor; C: Solvent C for TLC; Cl: Calculated; Lt: Literature; MW: Molecular weight; Ex: Experimental; N/A: Not apply; tr: Trace. All presumptively assigned chemical names were compared with available scientific literature and databases.
